# Supplementary material for: Structured random receptive fields enable informative sensory encodings
Source: PLoS Comput Biol. 2022 Oct 10;18(10):e1010484. doi: 10.1371/journal.pcbi.1010484 (PMC9584455; doi:10.1371/journal.pcbi.1010484)
Supplement: S1 Appendix — Fig A: Simulation results for the simplified frequency detection task. On the left, test error versus dataset size for kernel ridge regression using structured kernels (purple lines, by bandwidth) and the unstructured kernel (blue). On the right, test accuracy versus dataset size for SVM classifier readout trained on structured random features (purple lines, by bandwidth) and unstructured random features (blue). In both cases, task structural information improves performance, leading to less error. Fig B: Receptive fields of mechanosensory neurons. We show (A) biological receptive fields and (B) random samples from the fitted covariance model. Fig C: Covariance matrix of mechanosensory receptive fields and unstructured model. We compare the covariance matrices generated from the (A) receptive fields of 95 mechanosensory neurons, (B) unstructured GP model and (C) 95 random samples from the model. Fig D: Covariance matrix of mechanosensory receptive fields and the Fourier model (5). We compare the covariance matrices generated from the (A) receptive fields of 95 mechanosensory neurons, (B) Fourier GP model and (C) 95 random samples from the model. Fig E: Receptive fields from mechanosensory neurons, the unstructured model and the Fourier model (5). We show the receptive fields from the (A) mechanosensory neurons, (B) unstructured GP model and (C) the Fourier GP model. Fig F: Covariance matrix of V1 receptive fields and our model for white noise stimuli. We show the full structure of the covariance matrices, which are the 180 × 180 pixel region around the centers of these 504 × 504 pixel matrices. These matrices are generated from the (A) receptive fields of 8,358 mouse V1 neurons, (B) the GP model Eq (9), and (C) 8,358 random samples from the model. Fig G: Receptive fields of V1 neurons from white noise stimuli. We show (A) biological receptive fields and (B) random samples from the fitted covariance model. Fig H: Covariance matrix of V1 receptive fields and unstructu [file pcbi.1010484.s001.pdf]

# Supplementary information for: Structured random receptive fields enable informative sensory encodings

Biraj Pandey[1], Marius Pachitariu[2], Bingni W. Brunton[3], Kameron Decker Harris[4*]

**1** Department of Applied Mathematics, University of Washington, Seattle, Washington, United States of America
**2** Janelia Research Campus, Howard Hughes Medical Institute, Ashburn, Virginia, United States of America
**3** Department of Biology, University of Washington, Seattle, Washington, United States of America
**4** Department of Computer Science, Western Washington University, Seattle, Washington, United States of America

* kameron.harris@wwu.edu

# 1 Supplemental appendix

| Abbreviation | Meaning |
|---|---|
| ANN | artificial neural network |
| DCT | discrete cosine transform |
| DFT | discrete Fourier transform |
| DHT | discrete Hartley transform |
| GP | Gaussian process |
| LN | linear-nonlinear model of a neuron |
| ReLU | rectified linear unit, $\max(0, x)$ nonlinearity |
| RFN | random feature network |
| RKHS | reproducing kernel Hilbert space |
| SNR | signal-to-noise ratio |
| STA | spike triggered average |
| V1 | primary visual cortex |
| XOR | exclusive-or, boolean function |

Table A: List of abbreviations

| Symbol | Meaning |
|---|---|
| $\boldsymbol{x}$ | an input or stimulus to the network |
| $\boldsymbol{w}$ | input-hidden weights for a neuron |
| $\boldsymbol{\beta}, \beta_0$ | readout weights and offset |
| $y, \hat{y}$ | true and predicted output of the network |
| $d$ | dimension of the stimulus as a vector |
| $m$ | number of neurons in the hidden layer |
| $T$ | structured input space |
| $D$ | dimensions of input space $T$ |
| $L^2(T)$ | space of square-integrable functions over a domain $T$ |
| $\ell^2$ | vector space with norm $\|\boldsymbol{u}\| = \sqrt{\boldsymbol{u}^T\boldsymbol{u}}$ |
| $\|\cdot\|$ | the $L_2$ or $\ell_2$ norm for function or vector argument |
| $\|\cdot\|_F$ | the Frobenius norm of a matrix |
| $\langle a, b\rangle$ | the $L^2(T)$ inner product between functions, $\langle a, b\rangle = \int_{t\in T} a(t)b(t)\mathrm{d}t$ |
| $\boldsymbol{u}^T\boldsymbol{v}$ | finite-dimensional $\ell^2$ inner product, $\boldsymbol{u}^T\boldsymbol{v} = \sum_{i=1}^d u_i v_i$ |
| $\boldsymbol{I}_d$ | $d \times d$ identity matrix |
| $\boldsymbol{C}$ | $d \times d$ covariance matrix |
| $\mathcal{H}$ | RKHS, comes with inner product $\langle a, b\rangle_{\mathcal{H}}$ and norm $\|a\|_{\mathcal{H}} = \sqrt{\langle a, a\rangle_{\mathcal{H}}}$ |

Table B: List of important symbols

## 1.1 Function spaces for wide networks with structured receptive fields

RFNs are intimately connected to a popular class of supervised learning algorithms called kernel methods. As the network width grows, the inner product between the feature representations of two inputs $\boldsymbol{x}, \boldsymbol{x}'$ converges to a reproducing kernel

$$k(\boldsymbol{x}, \boldsymbol{x}') := \mathbb{E}_{\boldsymbol{w}}\left[h(\boldsymbol{x})h(\boldsymbol{x}')\right]. \tag{1}$$

The kernel defines a reproducing kernel Hilbert space (RKHS) of functions. The explicit form of the kernels corresponding to classical RFNs are known for several non-linear activation functions. For example, with the ReLU nonlinearity, no threshold, and unstructured Gaussian weights $\boldsymbol{w} \sim \mathcal{N}(0, \boldsymbol{I}_d)$, $k_{\mathrm{ReLU}}(\boldsymbol{x}, \boldsymbol{x}') = \frac{1}{\pi}\|\boldsymbol{x}\|\|\boldsymbol{x}'\|(\sin\theta + (\pi - \theta)\cos\theta)$ where $\theta = \arccos\left(\frac{\boldsymbol{x}^T\boldsymbol{x}'}{\|\boldsymbol{x}\|\|\boldsymbol{x}'\|}\right)$ [1].

We derive the kernel induced by our RFNs with hidden weights initialized from GPs. In this section we work in the discrete setting, but the continuous version is analogous. Recall the network equations that we use

$$\boldsymbol{h} = \sigma\left(\boldsymbol{W}\boldsymbol{x}\right), \qquad \hat{y} = \boldsymbol{\beta}^T\boldsymbol{h} + \beta_0, \tag{2}$$

and the basis change Theorem,

**Theorem 1** (Basis change formula). *Assume $\boldsymbol{w} \sim \mathcal{N}(0, \boldsymbol{C})$ with $\boldsymbol{C} = \boldsymbol{\Phi}\boldsymbol{\Lambda}^2\boldsymbol{\Phi}^T$ its eigenvalue decomposition. For $\boldsymbol{x} \in \mathbb{R}^d$, define*

$$\tilde{\boldsymbol{x}} := \boldsymbol{\Lambda}\boldsymbol{\Phi}^T\boldsymbol{x}. \tag{3}$$

*Then $\boldsymbol{w}^T\boldsymbol{x} = \boldsymbol{z}^T\tilde{\boldsymbol{x}}$ for $\boldsymbol{z} \sim \mathcal{N}(0, \boldsymbol{I}_d)$.*

By definition of the kernel Eq (1), network Eq (2), and Theorem 1, the kernel for structured features

$$\begin{aligned} k_{\text{struct}}(\boldsymbol{x}, \boldsymbol{x}') &= \mathbb{E}_{\boldsymbol{w}\sim\mathcal{N}(0,\boldsymbol{C})}\left[h(\boldsymbol{x})h(\boldsymbol{x}')\right] \\ &= \mathbb{E}_{\boldsymbol{w}\sim\mathcal{N}(0,\boldsymbol{C})}\left[\sigma(\boldsymbol{w}^T\boldsymbol{x})\sigma(\boldsymbol{w}^T\boldsymbol{x}')\right] \\ &= \mathbb{E}_{\boldsymbol{z}\sim\mathcal{N}(0,\boldsymbol{I}_d)}\left[\sigma\left(\boldsymbol{z}^T\tilde{\boldsymbol{x}}\right)\sigma\left(\boldsymbol{z}^T\tilde{\boldsymbol{x}}'\right)\right] \\ &:= k_{\text{unstruct}}(\tilde{\boldsymbol{x}}, \tilde{\boldsymbol{x}}'). \end{aligned} \tag{4}$$

Thus, the induced kernels from structured weights can be found in terms of unstructured weight kernels acting on the transformed inputs $\tilde{\boldsymbol{x}}$ and $\tilde{\boldsymbol{x}}'$. Taking ReLU as the nonlinearity for example, we get that $k_{\text{struct}}(\boldsymbol{x}, \boldsymbol{x}') = k_{\text{ReLU}}(\tilde{\boldsymbol{x}}, \tilde{\boldsymbol{x}}')$.

Every RKHS $\mathcal{H}$ comes with an inner product $\langle\cdot,\cdot\rangle_{\mathcal{H}}$ and norm $\|\cdot\|_{\mathcal{H}} = \sqrt{\langle\cdot,\cdot\rangle_{\mathcal{H}}}$. The norm and inner product can be expressed in terms of eigenvalues and eigenfunctions of the kernel itself, analogous to the eigendecomposition of the covariance function of the GP weights. Although it is beyond the scope of our paper to explain the theory in detail, there are well-established results showing that functions with small $\mathcal{H}$-norm are easier to learn than those with larger norm for a wide variety of kernel-based algorithms [2, 3]. In ridge regression, this effect is again equivalent to projection and filtering in the kernel eigenbasis, i.e. linear filtering in function space. Finally, end-to-end trained networks where the weights $\boldsymbol{W}$ are optimized may be studied with the related neural tangent kernel (NTK) when the step size is small [4]. The basis change Theorem 1 and Eq (4) give us a way to understand the RKHS of the stuctured network in terms of an unstructured network's RKHS acting on the transformed inputs $\tilde{\boldsymbol{x}}$.

### 1.1.1 Kernel eigenfunctions differ with structured weights

The structured RKHS has eigenfunctions which are different from the eigenfunctions of the unstructured RKHS. To see this, it's necessary to introduce a probability measure $\mu(\boldsymbol{x})$ for the data $\boldsymbol{x} \in \mathbb{R}^d$. Kernel learning is often understood [5] in the orthonormal basis for $L^2(\mu)$ given by the eigenfunctions $\psi_i$ of the integral operator $\mathcal{T}_k$ defined by

$$(\mathcal{T}_k f)(\boldsymbol{x}) = \int k(\boldsymbol{x}, \boldsymbol{x}')f(\boldsymbol{x}')\mathrm{d}\mu(\boldsymbol{x}').$$

A natural question to ask is, how does the eigensystems of $\mathcal{T}_{\text{struct}}$ and $\mathcal{T}_{\text{unstruct}}$ compare? The mapping from $\boldsymbol{x}$ to $\tilde{\boldsymbol{x}}$ induces a pushforward measure on $\tilde{\boldsymbol{x}}$ which we will call $\nu(\tilde{\boldsymbol{x}})$, and since the mapping is linear $\nu(\tilde{\boldsymbol{x}}) = \mu(\boldsymbol{\Phi}^T\boldsymbol{\Lambda}^{-1}\boldsymbol{x})$. (Note that $\mathrm{d}\nu(\boldsymbol{x}) = |\boldsymbol{\Lambda}|^{-1}\mathrm{d}\mu(\boldsymbol{\Phi}^T\boldsymbol{\Lambda}^{-1}\boldsymbol{x})$, and if $\mu$ is multivariate Gaussian, then $\nu$ is Gaussian with a different covariance.) Thus the integral operator $\mathcal{T}_{\text{struct}}$ under the data measure is equivalent to integrating kernel $k_{\text{unstruct}}$ under the pushforward measure. Because

$$\int k_{\text{unstruct}}(\boldsymbol{x}, \boldsymbol{x}')f(\boldsymbol{x}')\mathrm{d}\mu(\boldsymbol{x}') \qquad \text{and} \qquad \int k_{\text{unstruct}}(\boldsymbol{x}, \boldsymbol{x}')f(\boldsymbol{x}')\mathrm{d}\nu(\boldsymbol{x}')$$

are different, there is no general relationship that holds between the eigenfunctions. Different measures leading to different kernel eigenfunctions and eigenvalues can explain why structured weights have strong effects on learning, as shown by recent work demonstrating that areas of low input density are learned more slowly [6].

## 1.2 Kernel theory for frequency detection: a fully worked, highly nutritious, simplified example

To demonstrate how the kernel theory can also explain the benefits of structured random features, we developed a simplified frequency detection task. We fully explain the orthonormal basis, target function in that basis, kernels, and how the target function is harder to learn without knowledge of the structure. To our knowledge, this is the first technical result that explains how transforming the input data into an informative, lower-dimensional representation (essentially, preprocessing) leads to improved learning with kernel methods.

### 1.2.1 Data distribution and orthonormal basis

Frequency detection as presented in Appendix 1.7.1 is difficult to work with because of the different distributions of $x_+$ and $x_-$ samples. Kernel theory requires us to work with basis functions which are orthonormal with respect to the data measure, which in that case would be a mixture. To get around this issue, we simplify the data distribution so that all of our data are Gaussian white noise $\boldsymbol{x} \sim \mathcal{N}(0, \boldsymbol{I}_d)$. The Gaussian measure in 1-d has the natural orthogonal basis of (probabilist's) Hermite polynomials $He_k(x)$ [7]. The first few of these are

$$He_0(x) = 1, \quad He_1(x) = x, \quad He_2(x) = x^2 - 1.$$

We can normalize these as $\psi_k(x) = (k!)^{-1/2} He_k(x)$ so that orthonormality under the Gaussian distribution

$$\int_{-\infty}^{\infty} \psi_k(x)\psi_{k'}(x) \frac{1}{\sqrt{2\pi}} e^{-x^2/2} \mathrm{d}x = \delta_{k,k'} \tag{5}$$

is satisfied. To construct an orthonormal basis in $d > 1$ dimensions, we take the tensor product of Hermite polynomials,

$$\psi_{\boldsymbol{k}}(\boldsymbol{x}) = \prod_{i=1}^{d} \psi_{k_i}(x_i), \tag{6}$$

which is orthonormal for the multivariate Gaussian

$$\int_{\mathbb{R}^d} \psi_{\boldsymbol{k}}(\boldsymbol{x})\psi_{\boldsymbol{k}'}(\boldsymbol{x})(2\pi)^{-d/2} e^{-\|\boldsymbol{x}\|^2/2} \mathrm{d}\boldsymbol{x} = \delta_{\boldsymbol{k},\boldsymbol{k}'} \tag{7}$$

due to the separability of the integrals over each dimension.

### 1.2.2 Target function

The labels $y = f^*(\boldsymbol{x})$ are chosen to only depend on the amplitude of frequency $f_1$ in the white noise signal. Writing $\tilde{\boldsymbol{x}} = \boldsymbol{\Phi}^T \boldsymbol{x}$ for the input signal in frequency space, with $\boldsymbol{\Phi}$ the discrete cosine transform (DCT) matrix [8], the target function $f^*$ only depends on $|\tilde{x}_{f_1}|$. A simplified classfication task would then use

$$f^*(\boldsymbol{x}) = \begin{cases} +1 & \text{if } |\tilde{x}_{f_1}| \geq \theta \\ -1 & \text{if } |\tilde{x}_{f_1}| < \theta \end{cases}, \tag{8}$$

where the threshold $\theta$ is chosen so that 50% of the points are labeled $\pm 1$. However, we need to write the target function $f^*$ in the polynomial basis Eq (6), and Eq (8) is a step function which has an infinite polynomial expansion. A much simpler target function

$$f^*(\boldsymbol{x}) = |\tilde{x}_{f_1}|^2 - \theta^2 \tag{9}$$

still captures some of the behavior of Eq (8): It's negative when the power in frequency $f_1$ is below $\theta$ and positive above. We will analyze the quadratic surrogate Eq (9).

### 1.2.3 Kernel decomposition

We consider kernels $k_{\text{struct}}, k_{\text{unstruct}}$ of the form

$$\kappa(\boldsymbol{x}, \boldsymbol{x}') = \sum_{\boldsymbol{k}} \rho_{\boldsymbol{k}} \psi_{\boldsymbol{k}}(\boldsymbol{x}) \psi_{\boldsymbol{k}}(\boldsymbol{x}'), \tag{10}$$

where $\boldsymbol{k} = (k_1, k_2, \ldots, k_d)$ is a multi-index. We build $\kappa$ out of 1-d kernels

$$\kappa_i(x_i, x_i') = \sum_{k_i=0}^{\infty} \rho_{k_i} \psi_{k_i}(x_i) \psi_{k_i}(x_i'), \tag{11}$$

where the eigenvalue sequence $\rho_0, \rho_1, \ldots$ is shared across different $\kappa_i$. Each $\kappa_i$ defines an RKHS over functions of a single variable $\mathcal{H}_i$, and $\kappa(\boldsymbol{x}, \boldsymbol{x}') = \prod_{i=1}^{d} \kappa_i(x_i, x_i')$ defines an RKHS which is the tensor product $\mathcal{H} = \otimes_{i=1}^{d} \mathcal{H}_i$ with eigenvalues given as $\rho_{\boldsymbol{k}} = \prod_{i=1}^{d} \rho_{k_i}$. These tensor product polynomial kernels were considered in [9] for multiple kernel learning. We normalize the 1-d kernels Eq (11) so that $\sum_{k=0}^{\infty} \rho_k = 1$. This implies that

$$\sum_{\boldsymbol{k}} \rho_{\boldsymbol{k}} = \sum_{k_1, \ldots, k_d=0}^{\infty} \prod_{i=1}^{d} \rho_{k_i} = \left( \sum_{k_1=0}^{\infty} \rho_{k_1} \right) \cdots \left( \sum_{k_d=0}^{\infty} \rho_{k_d} \right) = 1, \tag{12}$$

by the separability of the eigenvalues, so that the $d$-dimensional kernel Eq (10) is also normalized. Furthermore, we can assume that all of the $\rho_i < 1$.

In general, random feature kernels will not be tensor products unless we make very specific choices of weights and nonlinearities. We only use the product structure for convenience with normalization. The form Eq (10) is, on the other hand, quite general and will hold for a variety of random feature kernels.

### 1.2.4 Target function in unstructured and structured networks

Now we analyze the target function in both unstructured and unstructured spaces. Using structured random feature map is equivalent to some deterministic remapping $\tilde{\boldsymbol{x}} = \boldsymbol{\Lambda} \boldsymbol{\Phi}^T \boldsymbol{x}$, where $\boldsymbol{\Phi}$ is an orthogonal matrix encompassing the basis change and $\boldsymbol{\Lambda}$ is a filtering matrix. Like in Section 1.8, let's use stationary bandpass features so that $\boldsymbol{\Lambda}$ just contains $d'$ entries which are equal to 1 with the rest 0, and $\boldsymbol{\Phi}$ is the DCT. Since the DCT is unitary, $\tilde{\boldsymbol{x}} \sim \mathcal{N}(0, I_{d'})$. We use the DCT rather than the DFT to avoid complications dealing with complex variables. Thus the transformed variables $\tilde{\boldsymbol{x}}$ and the original variables $\boldsymbol{x}$ both follow similar spherical Gaussian distributions just in different dimensions. This means that the Hermite polynomial basis Eq (6) is an orthonormal basis for both spaces.

Let $\tilde{\mathcal{H}}$ be the RKHS of functions after applying the transformation $\boldsymbol{x} \mapsto \tilde{\boldsymbol{x}}$. Then $\tilde{\mathcal{H}}$ has the kernel $\kappa(\tilde{\boldsymbol{x}}, \tilde{\boldsymbol{x}}')$ and eigenbasis $\psi_{\boldsymbol{k}}(\tilde{\boldsymbol{x}})$ under measure $\tilde{\boldsymbol{x}} \sim \mathcal{N}(0, I_{d'})$. We take $\mathcal{H}$ to be the RKHS of functions without ever transforming coordinates, i.e. the RKHS with kernel $\kappa(\boldsymbol{x}, \boldsymbol{x}')$ with eigenbasis $\psi_{\boldsymbol{k}}(\boldsymbol{x})$ under data measure $\boldsymbol{x} \sim \mathcal{N}(0, I_d)$.

The target function Eq (9) is simple to express in the eigenbasis of $\tilde{\mathcal{H}}$:

$$\begin{aligned} f^*(\tilde{\boldsymbol{x}}) &= |\tilde{x}_{f_1}|^2 - \theta^2 \\ &= He_2(\tilde{x}_{f_1}) - (\theta^2 - 1) He_0(\tilde{x}_{f_1}) \\ &= \sqrt{2} \psi_2(\tilde{x}_{f_1}) - (\theta^2 - 1) \psi_0(\tilde{x}_{f_1}). \end{aligned}$$

Any RKHS $\mathcal{F}$ with eigenvalues $\rho_i$ and eigenfunctions $\psi_i$ defines an inner-product

$$\langle f, g \rangle_{\mathcal{F}} = \sum_i \frac{\langle f, \psi_i \rangle_{L^2(\mu)} \langle g, \psi_i \rangle_{L^2}(\mu)}{\rho_i}, \tag{13}$$

where the $L^2(\mu)$ inner product is with respect to the data measure $\mu$ and $\|f\|_{\mathcal{F}}^2 = \langle f, f \rangle_{\mathcal{F}}$ [10]. The norm of our target function thus becomes

$$\|f^*\|_{\tilde{\mathcal{H}}}^2 = \frac{2}{\rho_2 \rho_0^{d'-1}} + \frac{(\theta^2 - 1)^2}{\rho_0^{d'}}. \tag{14}$$

We will see that working with the less informative RKHS $\mathcal{H}$ leads to a significantly larger norm.

To express the target function Eq (9) in the eigenbasis of $\mathcal{H}$ we must use the basis change matrix. Let $\boldsymbol{u}$ be the vector corresponding to row $f_1$ of $\boldsymbol{\Phi}^T$ so that $\tilde{x}_{f_1} = \boldsymbol{u}^T\boldsymbol{x} = \sum_{i=1}^d u_i x_i$. Thus,

$$\begin{aligned}
f^*(\boldsymbol{x}) &= |\tilde{x}_{f_1}|^2 - 1 \\
&= \left(\sum_{i=1}^d u_i x_i\right)^2 - \theta^2 \\
&= \sum_{i=1}^d u_i^2 x_i^2 + 2\sum_{i<j} u_i u_j x_i x_j - \theta^2 \\
&= \sum_{i=1}^d u_i^2(\sqrt{2}\psi_2(x_i) + 1) + 2\sum_{i<j} u_i u_j \psi_1(x_i)\psi_1(x_j) - \theta^2.
\end{aligned}$$

Reading off the coefficients of the basis terms (recall that $1 = \psi_{\mathbf{0}}(\boldsymbol{x})$), we get that

$$\begin{aligned}
\|f^*\|_{\mathcal{H}}^2 &= \frac{2}{\rho_2\rho_0^{d-1}}\left(\sum_{i=1}^d u_i^4\right) + \frac{1}{\rho_0^d}\left(\sum_{i=1}^d u_i^2 - \theta^2\right)^2 + \frac{4}{\rho_1^2\rho_0^{d-2}}\sum_{i<j}(u_i u_j)^2 \\
&= \frac{2}{\rho_2\rho_0^{d-1}}\left(\sum_{i=1}^d u_i^4\right) + \frac{(\theta^2-1)^2}{\rho_0^d} + \frac{4}{\rho_1^2\rho_0^{d-2}}\sum_{i<j}(u_i u_j)^2, \qquad (15)
\end{aligned}$$

where the $\sum_{i=1}^d u_i^2$ terms are equal to 1 because $\boldsymbol{\Phi}$ is unitary. Note that

$$1 = \|\boldsymbol{u}\boldsymbol{u}^T\|_F^2 = \sum_{i,j}(u_i u_j)^2 = 2\sum_{i<j}(u_i u_j)^2 + \sum_{i=1}^d u_i^4, \qquad (16)$$

so the terms involving sums combined are $O(\rho_0^d)$. Specifically for the DCT-II [8], components $u_i = \sqrt{\frac{2}{d}}\cos\left(\frac{\pi f_1}{d}\left(i - \frac{1}{2}\right)\right)$, so for any $f_1 \in \{1, \ldots (d-1)\}$

$$\sum_{i=1}^d u_i^4 = \frac{4}{d^2}\sum_{k=0}^{d-1}\cos^4\left(\frac{\pi f_1}{d}\left(k + \frac{1}{2}\right)\right) = \frac{3}{2d},$$

which implies that $\sum_{i<j}(u_i u_j)^2 = \frac{1}{2} - \frac{3}{4d}$. (For general unit vectors $\boldsymbol{u}$ we have that $1 \geq \sum_{i=1}^d u_i^4 \geq d^{-1}$ by Hölder's inequality.) Thus for DCT-II the norm is

$$\|f^*\|_{\mathcal{H}}^2 = \frac{3}{d\rho_2\rho_0^{d-1}} + \frac{(\theta^2-1)^2}{\rho_0^d} + \frac{2}{\rho_1^2\rho_0^{d-2}}\left(1 - \frac{3}{2d}\right). \qquad (17)$$

#### 1.2.5 Comparison of the learning performance in structured and unstructured RKHS

A very standard but rough bound on the generalization performance of kernel ridge regression or classification can be found by analyzing the Rademacher complexity of the class of linear functions in the RKHS [3, 2]. These upper bound the expected loss over new data in terms of the training loss plus an error term, c.f. Theorem 7.39 in [2] for kernel ridge regression. The error term controls the generalization gap between test and training losses and is typically proportional to the $\|f^*\|_{\mathcal{F}}$ (for 0/1 loss) or $\|f^*\|_{\mathcal{F}}^2$ (for square loss).[1] Thus, a function with small $\mathcal{F}$-norm is *easier to learn* in the *precise sense* that it takes a smaller training set size to achieve a given generalization gap when the norm is smaller.

We have computed $\|f^*\|_{\mathcal{H}}^2$ and $\|f^*\|_{\tilde{\mathcal{H}}}^2$ in Eq (15) and Eq (14). Examining those two expressions, we see that $\|f^*\|_{\mathcal{H}}^2 \gg \|f^*\|_{\tilde{\mathcal{H}}}^2$ due to two factors: First, there is the splitting of the quadratic target into $x_i^2$ and $x_i x_j$

[1] The trace of the kernel matrix also appears in these bounds, but this is unity due to normalization Eq (12).

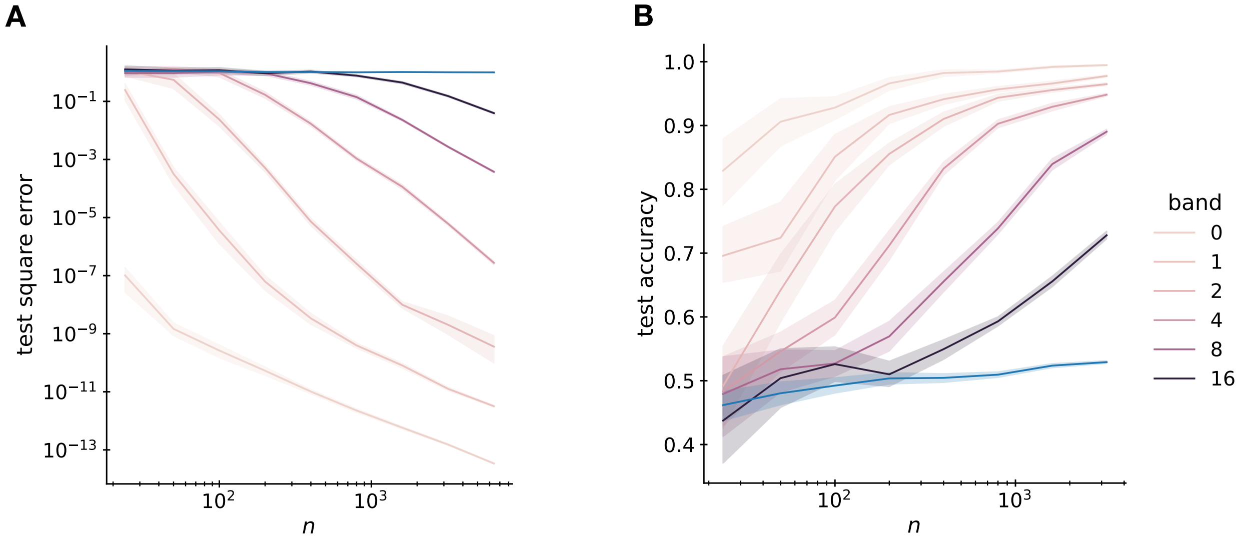


Figure A: **Simulation results for the simplified frequency detection task.** On the left, test error versus dataset size for kernel ridge regression using structured kernels (purple lines, by bandwidth) and the unstructured kernel (blue). On the right, test accuracy versus dataset size for SVM classifier readout trained on structured random features (purple lines, by bandwidth) and unstructured random features (blue). In both cases, task structural information improves performance, leading to less error and higher accuracy.

terms which creates the extra term $\frac{4}{\rho_1^2 \rho_0^{d-2}} \sum_{i<j} (u_i u_j)^2$ in the norm. This arises from *nonlinear effects* of the quadratic target function on the different components of the input signal. This nonlinear term is $O(\rho_0^{-d})$, the same order as the other terms, but Eq (15) will be larger than Eq (14) for $d' = d$ when $\rho_1^2 < \rho_0 \rho_2$. Secondly, the *dimension reduction* from $d$ to $d'$ dimensions means that analogous terms in Eq (15) and Eq (14) scale like $c^d$ and $c^{d'}$, respectively. This means that $\|f^*\|_{\mathcal{H}}^2 \geq c^{(d-d')} \|f^*\|_{\tilde{\mathcal{H}}}^2$. Note that the projection $\boldsymbol{x} \mapsto \tilde{\boldsymbol{x}}$ is a linear operation but has nonlinear consequences for the norm. The norm in the unstructured kernel space is exponentially larger than the norm in the structured kernel space.

Interpreting this exponential norm separation in light of the Rademacher generalization bounds, you would need to train on exponentially more samples with the unstructured versus the structured kernel to acheive the same bound. The majority of this effect is due to the dimension reduction factor, since the nonlinear factor only grows the norm by a constant. However, for target functions with contributions from many higher-order polynomials, the nonlinear factor would have a nonlinearly stronger effect. This is an interesting avenue for future research. More precise estimates of generalization performance are possible using theories such as [11]. However, the exponential gap between the performance of these two kernels is fundamental and not an artificial result from shortcomings of the Rademacher analysis.

#### 1.2.6 Simulation results support the theory

We ran simulations of the simplified frequency detection task with both kernel methods and random feature networks to check that this simplified task was similar to the task presented in the main text and Appendix 1.7.1. These simulations were performed with regression tasks with targets like Eq (9) and classification tasks equivalent to Eq (8). The results are consistent for a broad range of estimators and for both kernel and random feature networks.

For regression tasks, we used a tensor product kernel of the form Eq (10) with kernel ridge regression and the target function $f^*(\boldsymbol{x}) = a(|\tilde{x}_{f_1}|^2 - \theta^2)$. The constant $a$ was chosen so that the labels had standard deviation 1, meaning that a mean square error of 1 is equivalent to chance. For varying $n$ logarithmically spaced between 24 and 6,400 we generated training and testing sets of size $n/2$ in $d$ =1,000 dimensions with $f_1 = 16$. The training set was used to select the ridge parameter from powers of 10 in the range $[10^{-3}, \ldots, 10^3]$ by 5-fold cross-validation and error was computed on the test set. Each of these experiments

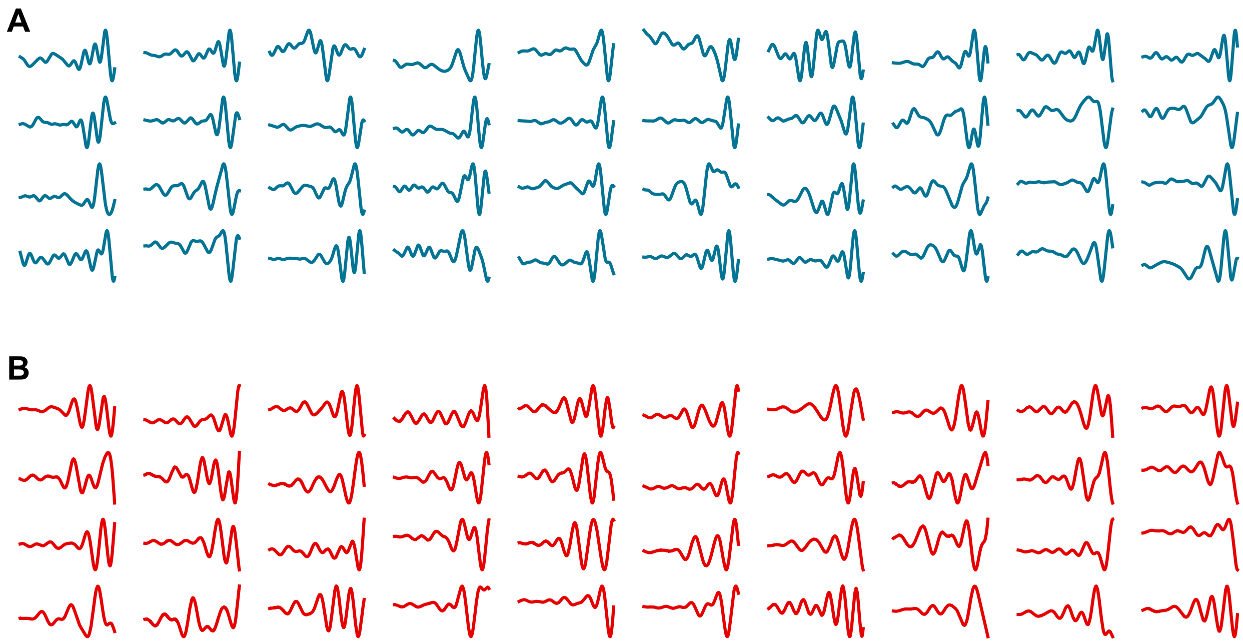


Figure B: **Receptive fields of mechanosensory neurons.** We show (A) biological receptive fields and (B) random samples from the fitted covariance model.

was repeated 20 times. We compare unstructured and structured kernels by either passing in the raw vectors $\boldsymbol{x}$ (unstructured) or first performing the DFT and passing in $(\tilde{\boldsymbol{x}})_{f_1-\texttt{band}:f_1+\texttt{band}}$. Here, `band` is a bandwidth parameter determining the number of components that are used in the structured kernel. When $\texttt{band} = 0$ we keep only the target frequency component.

The classification tasks were similar. We used the exact target function Eq (8), the range of $n$ from 24 to 3,200, same split into training and test sets, $d = 100$, and $f_1 = 16$. We classified using a random feature network with 2,000 neurons, ReLU nonlinearity, and zero bias trained with a linear SVM classifier readout. The SVM regularization strength was also swept over the range $[10^{-3}, \ldots, 10^{3}]$ by powers of 10 and selected via 5-fold cross-validation on the training set. The accuracy of the selected estimator was then computed on the test set. We compare both classical and mechanosensory receptive fields with bandwidth parameter `band` equivalent to $f_{\text{lo}} = f_1 - \texttt{band}$ and $f_{\text{hi}} = f_1 + \texttt{band}$ and no decay $(\gamma = \infty)$.

The results are shown in Fig. A. For either regression or classification, the best performance is acheived by the structured method with smallest bandwidth: error increases and accuracy degrades with bandwidth. The unstructured kernel and unstructured random feature methods both only perform at chance levels, square error $\approx 1$ or accuracy $\approx 0.5$. Similar results occur with kernel SVM on the classification task, kernel support vector regression, and for kernels which are not tensor product kernels (not shown). Furthermore, the random feature network's limiting kernel is an arc-cosine kernel which is not a tensor product kernel.

These simulations show that simplified frequency detection still exhibits the main features of the other tasks we study while remaining amenable to theoretical analysis. The simulation results are not sensitive to details of the experiment and are qualitatively similar when assumptions of the theory are broken. Getting quantitative predictions of training and testing errors from the theory is left to future work.

## 1.3 Covariance parameter optimization

Here we describe the details of how the GP covariances were fit to our various datasets.

### 1.3.1 Mechanosensor covariance

We aim to minimize the difference between the matrix generated by the covariance model $\boldsymbol{C}_{\text{model}}$ and the data $\boldsymbol{C}_{\text{data}}$, while keeping $f_{\text{lo}}$ smaller than $f_{\text{hi}}$. For simplicity, we measure the covariance mismatch with the

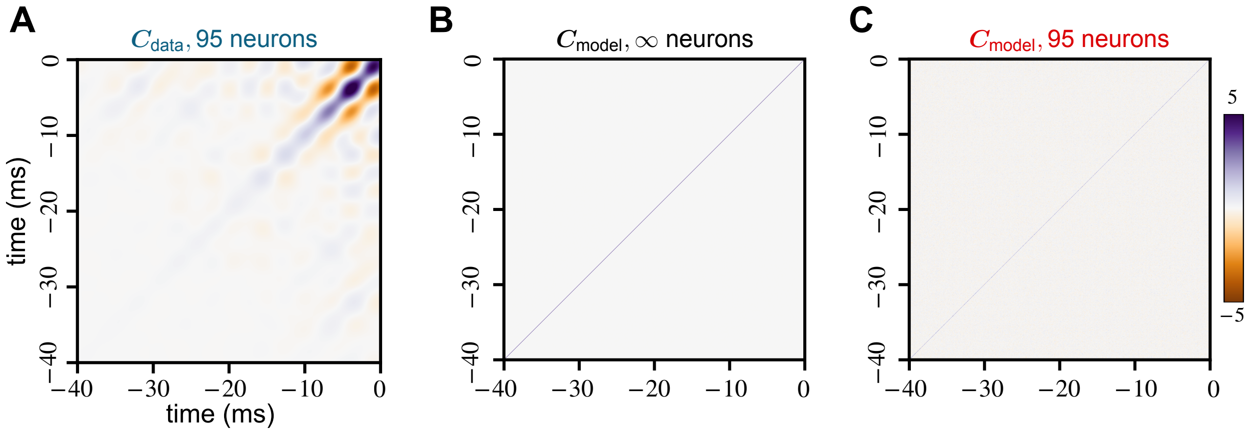


Figure C: **Covariance matrix of mechanosensory receptive fields and unstructured model.** We compare the covariance matrices generated from the (A) receptive fields of 95 mechanosensory neurons, (B) unstructured GP model and (C) 95 random samples from the model.

Frobenius norm, solving

$$\min_{f_{\rm lo}, f_{\rm hi}, \gamma} \|\boldsymbol{C}_{\rm model}(f_{\rm lo}, f_{\rm hi}, \gamma) - \boldsymbol{C}_{\rm data}\|_F \qquad (18)$$
$$\text{subject to: } f_{\rm hi} \geq f_{\rm lo}.$$

We use the trust region algorithm provided by the `scipy.optimize.minimize` to solve Eq (18).

### 1.3.2 V1 covariance

To fit the covariance model to the data, we formulate an optimization problem over the model parameters $s$ and $f$, where we minimize the Frobenius norm of the difference between the covariance matrix $\boldsymbol{C}_{\rm model}$ and $\boldsymbol{C}_{\rm data}$:

$$\min_{s,f} \|\boldsymbol{C}_{\rm model}(s, f) - \boldsymbol{C}_{\rm data}\|_F. \qquad (19)$$

We solve Eq (19) using the Broyden–Fletcher–Goldfarb–Shannon (BFGS) algorithm provided by the `scipy.optimize.minimize` package.

## 1.4 Null receptive field models

We construct null GP models for both mechanosensors and V1 for comparison.

### 1.4.1 Mechanosensor covariance

We compare the data covariance matrix with the unstructured model and the Fourier model:

$$C(t, t') = \overbrace{\sum_{k=0}^{\infty} \lambda_k^2 \cos\left(\omega_k (t - t')\right)}^{\text{stationary process}}. \qquad (20)$$

For the Fourier model, we fit the $f_{hi}$ and $f_{lo}$ parameters to data by minimizing the Frobenius error in the covariance matrix, finding $f_{lo} = 75$ Hz, $f_{hi} = 200$ Hz. The resulting covariance matrices are shown in Fig. C and Fig. D. Receptive field samples from the null models are shown in Fig. E. The samples from the Fourier model are smooth but do not decay in time like biological receptive fields.

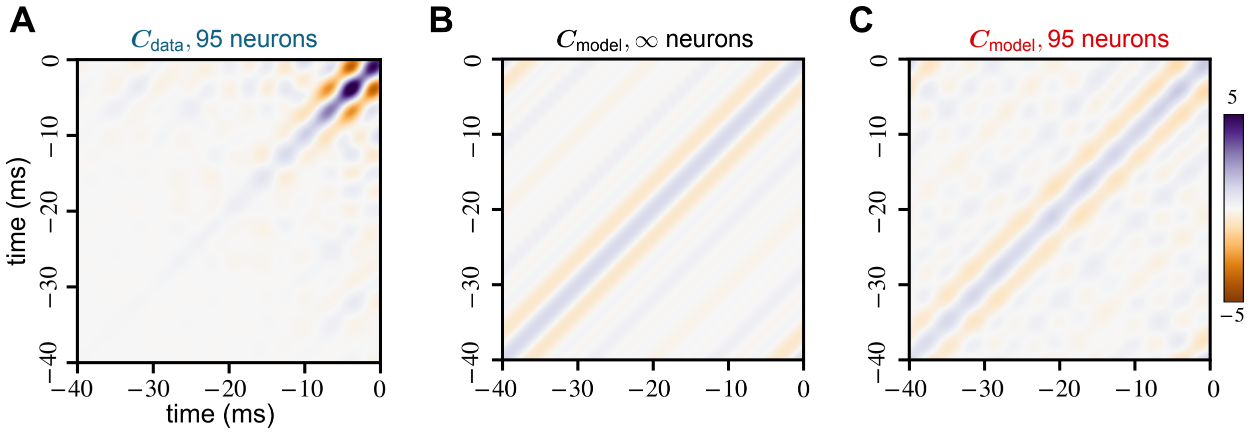


Figure D: **Covariance matrix of mechanosensory receptive fields and the Fourier model (20).** We compare the covariance matrices generated from the (A) receptive fields of 95 mechanosensory neurons, (B) Fourier GP model and (C) 95 random samples from the model.

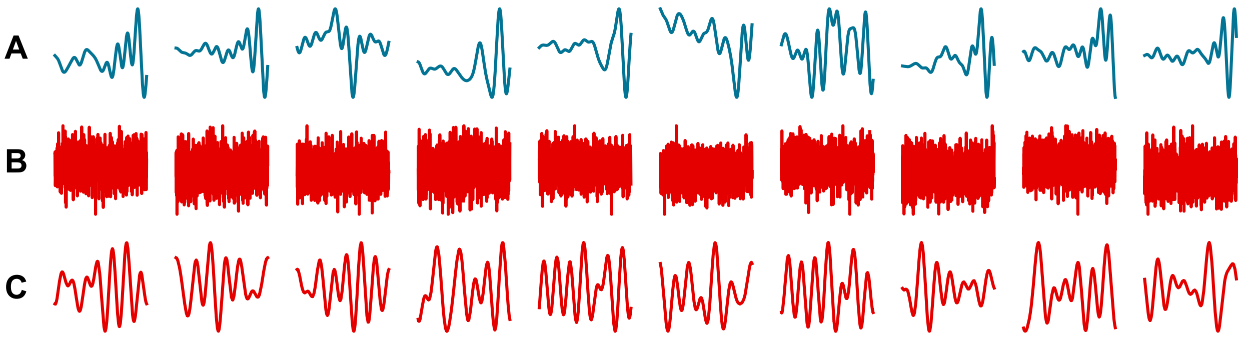


Figure E: **Receptive fields from mechanosensory neurons, the unstructured model and the Fourier model (20).** We show the receptive fields from the (A) mechanosensory neurons, (B) unstructured GP model and (C) the Fourier GP model.

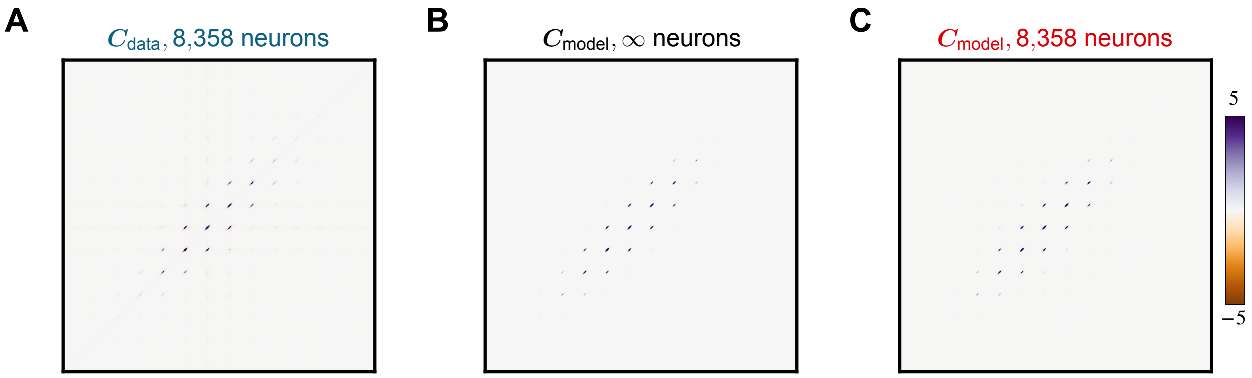


Figure F: **Covariance matrix of V1 receptive fields and our model for white noise stimuli.** We show the full structure of the covariance matrices, which are the $180 \times 180$ pixel region around the centers of these $504 \times 504$ pixel matrices. .These matrices are generated from the (A) receptive fields of 8,358 mouse V1 neurons, (B) the GP model Eq (21), and (C) 8,358 random samples from the model.

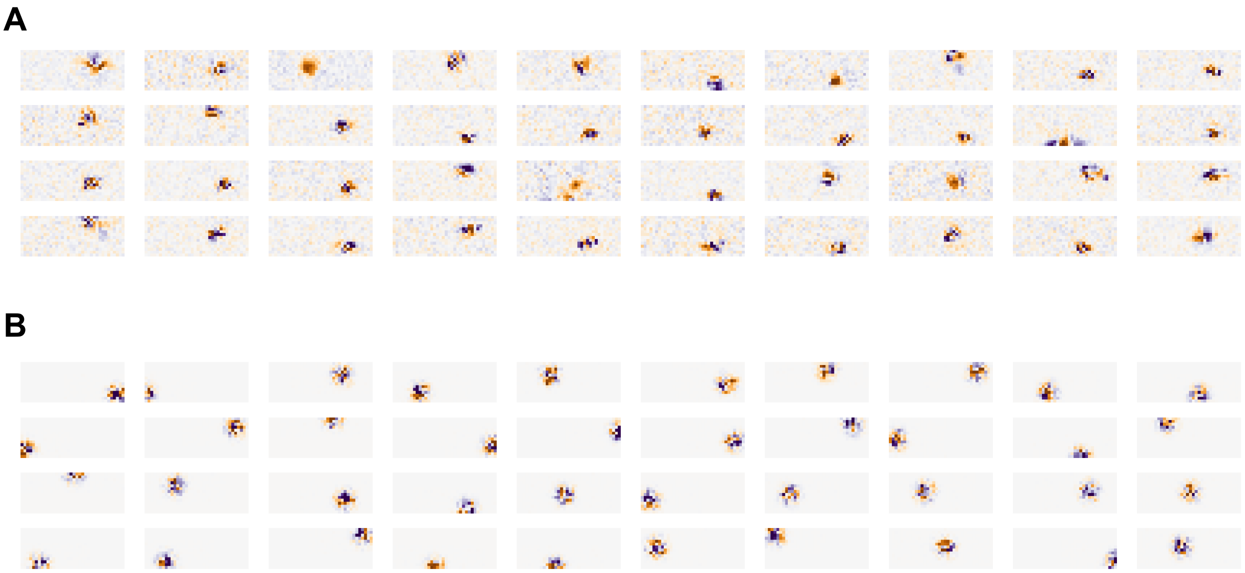


Figure G: **Receptive fields of V1 neurons from white noise stimuli.** We show (A) biological receptive fields and (B) random samples from the fitted covariance model.

### 1.4.2 V1 covariance

We compare the data covariance matrix with the unstructured model and the translation invariant version of the V1 model. Recall that the localized V1 model had covariance

$$C(\boldsymbol{t}, \boldsymbol{t}') = \overbrace{\exp\left(-\frac{\|\boldsymbol{t} - \boldsymbol{t}'\|^2}{2f^2}\right)}^{\text{smooth receptive fields}} \cdot \overbrace{\exp\left(-\frac{\|\boldsymbol{t} - \boldsymbol{c}\|^2 + \|\boldsymbol{t}' - \boldsymbol{c}\|^2}{2s^2}\right)}^{\text{localized to a center } c}. \tag{21}$$

For the translation invariant model, we remove the localizing exponential and only fit the spatial frequency parameter, $f$ (finding $f = 0.73$ pixels). The neuron weights are generated from a covariance function of the following form:

$$C(\boldsymbol{t}, \boldsymbol{t}') = \overbrace{\exp\left(-\frac{\|\boldsymbol{t} - \boldsymbol{t}'\|^2}{2f^2}\right)}^{\text{smooth receptive fields}} \tag{22}$$

The resulting covariance matrices are shown in Fig. H and Fig. I. Receptive field samples from the null models are show in Fig. J.

## 1.5 Derivation of eigenfunctions of V1 covariance function

The covariance between two pixel locations $\boldsymbol{t} = (t_1, t_2), \boldsymbol{t}' = (t_1', t_2') \in \boldsymbol{R}^2$ is given by

$$\begin{aligned} C(\boldsymbol{t}, \boldsymbol{t}') &= e^{-\frac{\|\boldsymbol{t}-\boldsymbol{t}'\|^2}{2f^2}} e^{-\frac{\|\boldsymbol{t}\|^2 + \|\boldsymbol{t}'\|^2}{2s^2}} \\ &= e^{-\frac{(t_1 - t_1')^2}{2f^2}} e^{-\frac{(t_1^2 + t_1'^2)}{2s^2}} \cdot e^{-\frac{(t_2 - t_2')^2}{2f^2}} e^{-\frac{(t_2^2 + t_2'^2)}{2s^2}} \\ &= \prod_{i=1}^{2} e^{-\alpha(t_i - t_i')^2} e^{-\delta(t_i^2 + t_i'^2)} \qquad \text{for } \alpha := \frac{1}{2f^2},\ \delta := \frac{1}{2s^2}. \end{aligned}$$

Since covariance function factors into a product of functions of variables $t_1, t_1'$ and $t_2, t_2'$, the multidimensional eigenfunctions $\phi_{\boldsymbol{k}}(\boldsymbol{t})$ and eigenvalues $\lambda_{\boldsymbol{k}}^2$ also factor into a product of 1-dimensional eigenfunction and

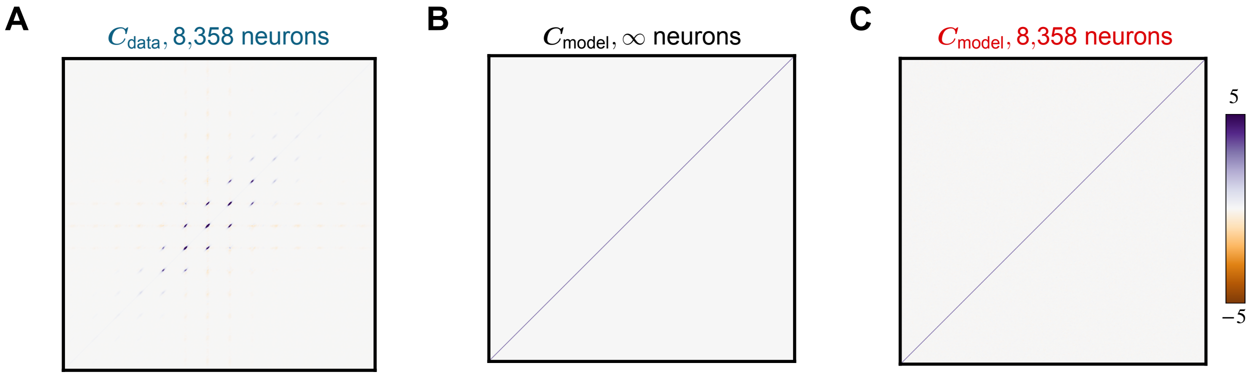


Figure H: **Covariance matrix of V1 receptive fields and unstructured model for white noise stimuli.** We compare the covariance matrices generated from the (A) receptive fields of 8,358 mice V1 neurons, (B) unstructured GP model and (C) 8,358 random samples from the model.

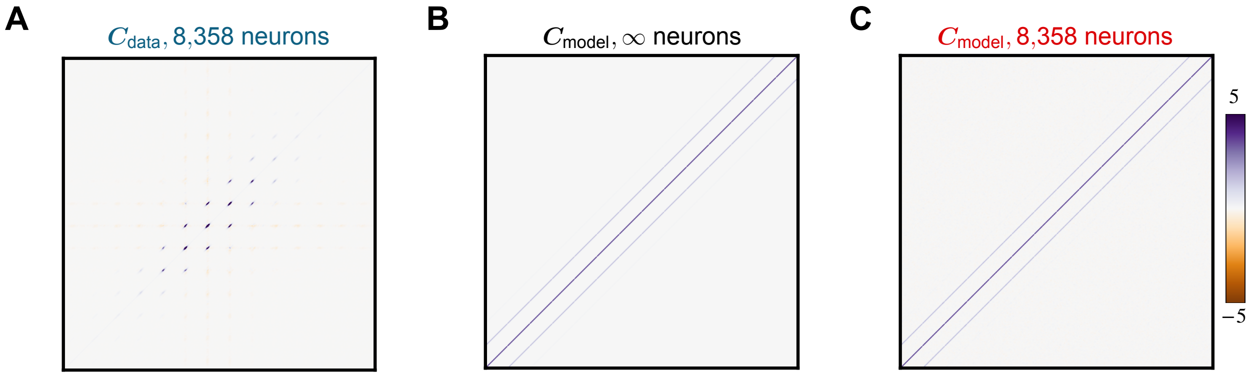


Figure I: **Covariance matrix of V1 receptive fields and translation invariant V1 model (Eq. 22) for white noise stimuli.** We compare the covariance matrices generated from the (A) receptive fields of 8,358 mice V1 neurons, (B) translation invariant version of the V1 GP model and (C) 8,358 random samples from the model.

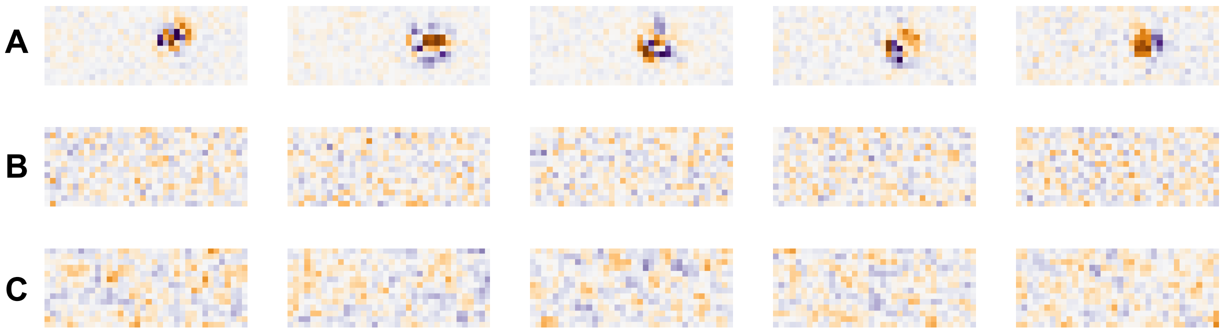


Figure J: **Receptive fields from V1 neurons, the unstructured model and the translation invariant V1 model (22).** We show the receptive fields from the (A) V1 neurons, (B) unstructured GP model and (C) the translation invariant V1 GP model.

eigenvalues, i.e. $\phi_{\boldsymbol{k}}(\boldsymbol{t}) = \prod_{i=1}^{2} \phi_{k_i}(t_i)$ and $\lambda_{\boldsymbol{k}}^2 = \prod_{i=1} \lambda_{k_i}^2$. This holds for $d > 2$ dimensions as well. So we

work in 1-d and search for eigenfunctions and eigenvalues such that,

$$\int_{-\infty}^{\infty} C(t,t')\phi_k(t)dt = \lambda_k^2 \phi_k(t'),$$

with $C(t,t') = e^{-\alpha(t-t')^2} e^{-\delta(t^2+t'^2)}$.

We make the ansatz that $\phi_k(t) = e^{-c_1 t^2} H_k(c_2 t)$, where $H_k$ is the $k^{th}$ Hermite polynomial (physicists' convention) [12] and $c_1, c_2$ are constants. With this guess for the eigenfunctions,

$$\begin{aligned}
\int_{-\infty}^{\infty} C(t,t')\phi_k(t)dt &= \int_{-\infty}^{\infty} e^{-\alpha(t-t')^2} e^{-\delta(t^2+t'^2)} e^{-c_1 t^2} H_k(c_2 t)dt \\
&= \int_{-\infty}^{\infty} e^{-Xt^2+Yt-t'^2(\alpha+\delta)} H_k(c_2 t)dt && (X := \alpha+\delta+c_1, Y := 2\alpha t') \\
&= e^{-t'^2(\alpha+\delta)+\frac{Y^2}{4X}} \int_{-\infty}^{\infty} e^{-(\sqrt{X}t-\frac{Y}{2\sqrt{X}})^2} H_k(c_2 t)dt && \text{(completing the square)} \\
&= \frac{1}{\sqrt{X}} e^{-t'^2(\alpha+\delta)+\frac{Y^2}{4X}} \int_{-\infty}^{\infty} e^{-\left(u-\frac{Y}{2\sqrt{X}}\right)^2} H_k(u\frac{c_2}{\sqrt{X}}) && (u = \sqrt{X}t) \\
&= \underbrace{\sqrt{\frac{\pi}{X}}\left(1-\frac{c_2^2}{X}\right)^{k/2}}_{\lambda_k^2} \underbrace{e^{-t'^2(\alpha+\delta-\frac{\alpha^2}{X})} H_k\left(\frac{c_2\alpha}{X(1-\frac{c_2^2}{X})^{1/2}}t'\right)}_{\hat{\phi}_k(t')}. && ([12], 7.374.8)
\end{aligned}$$

Solving for the unknown constants leads to the equations

$$\begin{aligned}
c_1 &= \alpha+\delta-\frac{\alpha^2}{X} && \implies c_1 = \sqrt{\delta(2\alpha+\delta)}, \\
c_2 &= \frac{c_2\alpha}{X\left(1-\frac{c_2^2}{X}\right)^{1/2}} && \implies c_2 = \sqrt{(\alpha+\delta+c_1)\left(1-\frac{\alpha^2}{(\alpha+\delta+c_1)}\right)} = \sqrt{2c_1}.
\end{aligned}$$

The last step is to find the normalization constant for the eigenfunctions:

$$\begin{aligned}
\int_{-\infty}^{\infty} |\hat{\phi}_k(t)|^2 dt &= \int_{\infty}^{\infty} e^{-2c_1 t^2} H_k^2(c_2 t)dt \\
&= \frac{1}{c_2}\int_{\infty}^{\infty} e^{-u^2} H_k^2(u)du && (u = c_2 t,\ c_2^2 = 2c_1) \\
&= \frac{2^k k! \sqrt{\pi}}{c_2}. && ([12], 7.374.1)
\end{aligned}$$

Therefore, our orthonormal eigenfunctions and eigenvalues for the 1-dimensional covariance are

$$\phi_k(t) = \frac{c_2}{2^k k! \sqrt{\pi}} e^{-c_1 t^2} H_k(c_2 t), \qquad \lambda_k^2 = \sqrt{\frac{\pi}{\alpha+\delta+c_1}}\left(1-\frac{c_2^2}{\alpha+\delta+c_1}\right)^{k/2}, \tag{23}$$

where $c_1 = \sqrt{\delta(2\alpha+\delta)}, c_2 = \sqrt{2c_1}$. Note that $\lambda_k^2 \propto c_3^k$ with $c_3 = \sqrt{1-\frac{c^2}{\alpha+\delta+c_1}}$, so that the spectrum decays exponentially.

## 1.6 Distributed receptive field centers imply a sum kernel space

To generate our V1-inspired weights, we first sample a center $\boldsymbol{c}$ uniformly at random from the pixels in the image; call this set of pixels $S$. We will now derive the kernel for this weight sampling.

Suppose that all of the weights are sampled with a single center $\boldsymbol{c}$. Then Eq (4) tells us that the structured kernel associated with the RFN

$$\begin{aligned}
k_{\text{struct}}(\boldsymbol{x}, \boldsymbol{x}'; \boldsymbol{c}) &= k_{\text{unstruct}}(\boldsymbol{\Lambda_c}\boldsymbol{\Phi_c}^T\boldsymbol{x}, \boldsymbol{\Lambda_c}\boldsymbol{\Phi_c}^T\boldsymbol{x}') \\
&= k_{\text{unstruct}}(\tilde{\boldsymbol{x}}_{\boldsymbol{c}}, \tilde{\boldsymbol{x}}'_{\boldsymbol{c}}),
\end{aligned} \tag{24}$$

where we have defined the *local basis change*

$$\tilde{\boldsymbol{x}}_{\boldsymbol{c}} = \boldsymbol{\Lambda}_{\boldsymbol{c}} \boldsymbol{\Phi}_{\boldsymbol{c}}^T \boldsymbol{x}. \tag{25}$$

This local basis change projects into a basis of Hermite wavelets $\boldsymbol{\Phi}_{\boldsymbol{c}}$ centered at $\boldsymbol{c}$ and filters according to the eigenvalues $\boldsymbol{\Lambda}_{\boldsymbol{c}}$. The reproducing kernel Eq (24) defines an RKHS of functions $\mathcal{H}_{\boldsymbol{c}}$ which take images as their input and produce a real-valued output. The RKHS is a Hilbert space and thus has a norm $\| \cdot \|_{\mathcal{H}_{\boldsymbol{c}}}$. Functions with small $\mathcal{H}_{\boldsymbol{c}}$-norm are, informally, smooth functions of the local wavelet coefficients $\tilde{\boldsymbol{x}}_{\boldsymbol{c}}$.

In our experiments, we actually sample weights from all centers $\boldsymbol{c} \in S$ with equal probability. Taking the expectation over the centers, this means that the kernel will be an average over all of the local kernels Eq (24),

$$k_{\text{struct}}(\boldsymbol{x}, \boldsymbol{x}') = \frac{1}{|S|} \sum_{\boldsymbol{c} \in S} k_{\text{struct}}(\boldsymbol{x}, \boldsymbol{x}'; \boldsymbol{c}). \tag{26}$$

Let $\mathcal{H}$ be the RKHS associated with $k_{\text{struct}}(\cdot, \cdot)$, another space of functions that take in images and output a real number. The sum Eq (26) implies that $\mathcal{H} = \bigoplus_{\boldsymbol{c} \in S} \mathcal{H}_{\boldsymbol{c}}$, i.e. the RKHS is a direct sum of local RKHS's [2]. This means that any function $f \in \mathcal{H}$ can be written as $f = \sum_{\boldsymbol{c} \in S} f_{\boldsymbol{c}}$, with every $f_{\boldsymbol{c}} \in \mathcal{H}_{\boldsymbol{c}}$. The norm of this function comes from taking a minimum over all such decompositions

$$\|f\|_{\mathcal{H}} = \min_{f_{\boldsymbol{c}} : f = \sum_{\boldsymbol{c} \in S} f_{\boldsymbol{c}}} \sqrt{|S| \sum_{\boldsymbol{c} \in S} \|f_{\boldsymbol{c}}\|^2_{\mathcal{H}_{\boldsymbol{c}}}}.$$

We can think of functions with small $\mathcal{H}$-norm, which will be easiest to learn, as sums of smooth functions of local wavelet coefficients. This is equivalent to using convolutions, i.e. [13].

## 1.7 Timeseries data generation

We detail how the two frequency classification tasks from (detection and XOR) are generated. In both tasks, each example is an $L$ ms timeseries sampled at $f$ Hz, making each $\boldsymbol{x}$ a vector of length $d = L \times f$. Thus in the discrete setting, we only have $d$ total frequencies. While the math below show continuous signals, in our code we generate analagous discrete signals using the discrete Fourier transform basis.

### 1.7.1 Frequency detection

The frequency detection task is a binary classification task. The positive examples contain a pure sinusoidal signal with frequency $f_1$ and additive Gaussian noise. The negative examples are just white noise. They are generated in the following way:

$$x_+(t) = \overbrace{a \sqrt{\frac{2}{L}} \left(\eta_f \cos(f_1 t) - \xi_f \sin(f_1 t)\right)}^{\text{pure frequency}} + \overbrace{\sqrt{1-a^2} \sqrt{\frac{2}{(d-1)L}} \sum_{\substack{j=0 \\ j: \omega_j \neq f_1}}^{d-1} \left(\eta_j \cos(\omega_j t) - \xi_j \sin(\omega_j t)\right)}^{\text{additive Gaussian noise}}$$

$$x_-(t) = \sqrt{\frac{2}{dL}} \sum_{j=0}^{d-1} \left(\eta_j \cos(\omega_j t) - \xi_j \sin(\omega_j t)\right)$$

where $\omega_j = 2\pi j / L$ is the $j$-th natural frequency, $a$ is a parameter that sets the SNR, and the coefficients $\eta_j, \xi_j, \eta_f, \xi_f$ are random variables uniformly sampled from the unit circle (which gives each frequency component a random phase).

We define

$$\text{SNR} := \frac{a^2}{1 - a^2}, \tag{27}$$

with $a \in [0, 1]$. Larger $a$ means a larger contribution of the pure tone and smaller amplitude noise. Note that $\|x_-(t)\|^2_{L^2([0,L])} = \|x_+(t)\|^2_{L^2([0,L])} = 1$. The generation process ensures that the $L^2$ energy of both the

negative and positive examples are matched and that the SNR is equal to the ratio of energy captured in frequency $f_{\text{lo}}$ to the total energy in all other components.

We generate a balanced dataset with 7,000 timeseries signals which we split into a training set with 5,600 examples and a test set with 1,400 examples. We tuned the GP covariance parameters $f_{\text{lo}}$, $f_{\text{hi}}$, and $\gamma$ from

$$C(t,t') = \overbrace{\exp\left(-\frac{(t+t')}{\gamma}\right)}^{\text{localized}} \overbrace{\sum_{k=0}^{\infty} \lambda_k^2 \cos\left(\omega_k(t-t')\right)}^{\text{stationary process}}, \qquad \lambda_k = \overbrace{\begin{cases} 1 & f_{\text{lo}} \le \omega_k \le f_{\text{hi}} \\ 0 & \text{otherwise} \end{cases}}^{\text{bandlimited, flat-power spectrum}}, \tag{28}$$

using 3-fold cross validation on the training set. We found the optimal parameters for a network with 20 hidden neurons and used them for all hidden layer widths. We tested $f_{\text{lo}}$ and $f_{\text{hi}}$ parameters from 10 Hz to 200 Hz at increments of 10 Hz. For $\gamma$, we set the parameter range to be from 10 ms to 100 ms and used all parameters at increments of 10 ms. Using grid search, we tested all combinations of these parameters. The optimal model was refit using all training 5,600 samples, and the errors we report were measured on the test set.

### 1.7.2 Frequency XOR

We use a similar set up to generate the timeseries for the frequency exclusive-or (XOR) task. The positive examples are either frequency $f_1$ or $f_2$ Hz pure sinusoids with additive Gaussian noise. The negative examples are either mixed frequency timeseries (with both $f_1$ and $f_2$ Hz signals) or pure Gaussian noise. They are generated in the following way:

$$x_{+,k}(t) = \overbrace{a\sqrt{\frac{2}{L}}\left(\eta_f \cos(f_k t) - \xi_f \sin(f_k t)\right)}^{\text{pure frequency}} + \overbrace{\sqrt{\frac{2(1-a^2)}{(d-1)L}} \sum_{\substack{j=0 \\ j:\omega_j \neq f_j}}^{d-1} \left(\eta_j \cos(\omega_j t) - \xi_j \sin(\omega_j t)\right)}^{\text{additive Gaussian noise}}$$

$$x_{-,\text{noise}}(t) = \sqrt{\frac{2}{dL}} \sum_{j=0}^{d-1} \left(\eta_j \cos(\omega_j t) - \xi_j \sin(\omega_j t)\right)$$

$$x_{-,\text{mixed}}(t) = \frac{a}{\sqrt{L}} \sum_{j\in\{1,2\}} \left(\eta_j \cos(f_j t) - \xi_j \sin(f_j t)\right) + \sqrt{\frac{2(1-a^2)}{(d-2)L}} \sum_{\substack{j=0 \\ j:\omega_j \neq f_1, f_2}}^{d-1} \left(\eta_j \cos(\omega_j t) - \xi_j \sin(\omega_j t)\right),$$

for $k \in \{1, 2\}$ in the $x_{+,k}(t)$ function. The constants, random variables, and details of SNR are identical to the frequency detection section. The datasets we generate have balanced proportions of $x_{+,1}$, $x_{+,2}$, $x_{-,\text{noise}}$, and $x_{-,\text{mixed}}$ signals.

## 1.8 SNR amplification via filtering

Let's consider the simple frequency detection task with stationary bandpass features. Since these features are stationary, their eigenvectors are Fourier modes, i.e. $\mathbf{\Phi}$ is the discrete Fourier transform (DFT) matrix. Assume the features encode a bandpass filter, which means that $\mathbf{\Lambda} = \text{diag}(\lambda_i)$, $i \in \{0, \ldots, d-1\}$, with $\lambda_i = 1$ for $i_{\text{lo}} \le i \le i_{\text{hi}}$ and 0 otherwise.

In frequency detection, a single frequency component (discrete Fourier mode) contains the signal with energy $a^2$. The other $d-1$ components each have energy $\frac{1-a^2}{d-1}$, for a total energy of $1-a^2$ contained in this white noise. After the basis change is applied to any input $\boldsymbol{x}$, the transformed vector $\tilde{\boldsymbol{x}} = \mathbf{\Lambda}\mathbf{\Phi}^*\boldsymbol{x}$ will have zeros in all entries outside the passband. (We use the conjugate transpose $\mathbf{\Phi}^*$ here rather than the transpose since the DFT matrix is complex; the interpretation is the same.) This makes the new representation $\tilde{\boldsymbol{x}}$ effectively $d'$-dimensional, where $d' = i_{\text{hi}} - i_{\text{lo}}$.

Now, first assume that the signal is within the passband. The total noise energy in the transformed representation becomes $(1-a^2)\frac{d'-1}{d-1}$, since one of the $d'$ components is still signal, and no energy is lost in the retained components because $\mathbf{\Phi}$ is unitary. The overall noise is shrunk by a factor of $\frac{d'-1}{d-1}$, so the SNR

gets boosted by $\frac{d-1}{d'-1}$. In the limiting case where $d' = 1$, the noise energy is 0 and SNR is infinite. On the other hand, if the signal lies outside the passband the SNR is reduced to 0.

## 1.9 Implementation details and code availability

In all experiments with RFNs, the training algorithm is an SVM classifier with squared hinge loss provided by the `sklearn.svm.LinearSVC` package and all other parameters set to their defaults. We used `scipy` [14] and `numpy` [15] to construct both classical unstructured and neural-inspired structured weights. For the experiments with fully-trained networks, we used `pytorch` [16]. The cross entropy loss function was optimized using full batch stochastic gradient gescent (SGD) optimizer, i.e. gradient descent (GD). Our code is available at `https://github.com/BruntonUWBio/structured-random-features`.

## 1.10 Covariance of V1 neurons with other stimuli

We repeat the covariance analysis of the V1-inspired weights on three additional datasets of V1 neurons. Different stimuli were shown in each dataset to calculate the receptive field.

The first dataset was provided by Ringach et al. from their work on characterizing the spatial structure of simple receptive fields in macaque (*Macaca fascicularis*) V1 [17]. The spikes of 250 neurons were recorded in response to drifting sinusoidal gratings. The receptive fields were calculated from the stimuli and responses using subspace reverse correlation. Because of the bandlimited properties of sinusoidal stimuli, this experiment biases the reconstruction towards smooth receptive fields. The receptive fields were of various sizes: 32 pixels $\times$ 32 pixels, 64 pixels $\times$ 64 pixels, and 128 pixels $\times$ 128 pixels. We resized them to a common dimension of 32 pixels $\times$ 32 pixels using local mean averaging. We find the optimal covariance parameters that fit the data to be $s = 2.41$ and $f = 0.95$ pixels. The covariance matrices and eigenfunctions are shown in Fig. K. Examples of biological receptive fields and random samples from the fitted model are shown in Fig. L in the Appendix.

The second dataset contains the responses of 69,957 neurons recorded from the primary visual cortex of mice bred to express GCaMP6s. We presented 5,000 static natural images of 24 $\times$ 27 pixels in random order for 3 trials each. We calculated the receptive fields from the natural images and calcium responses of cells using ridge regression with an $\ell^2$ penalty set to 0.1 after each image pixel was z-scored across images. We used the average receptive field over all three trials. For the covariance analysis, we picked cells with SNR $> 0.4$. This gave us 10,782 cells. The optimal covariance parameters that fit the data are $s = 5.40$ and $f = 1.17$ pixels. Examples of biological receptive fields and random samples from the model are shown in Fig. N. The covariance matrices and eigenfunctions are shown in Fig. M. Examples of biological receptive fields and random samples from the fitted model are shown in Fig. N. Repeating this analysis using receptive fields from individual trials yields identical results.

The third dataset contains the responses of 4,337 neurons also recorded from the primary visual cortex of mice bred to express GCaMP6s. The mice were shown static discrete Hartley transform (DHT, similar to a real-valued discrete Fourier transform) basis functions of size 30 $\times$ 80 pixels, and the calcium responses of neurons were recorded. The receptive fields were calculated using ridge regression without any $\ell^2$ penalty. Here, we picked cells with SNR $> 1$ for analysis. We were left with 2,698 cells. The optimal covariance parameters that fit the data are $s = 10.46$ and $f = 1.20$ pixels. The covariance matrices and eigenfunctions are shown in Fig. O. Examples of biological receptive fields and random samples from the fitted model are shown in Fig. P.

## 1.11 Initialization of networks with structured weights

We show results of initializing fully trained neural networks across a range of network widths (50, 100, 400, and 1,000) and learning rates ($10^{-3}$, $10^{-2}$, and $10^{-1}$) in Figures Q, R, S, and T.

## 1.12 Deep network experiments

We experimented with using the V1-inspired weight initialization in the first two convolutional layers of AlexNet [18] and training on the ImageNet Large Scale Visual Recognition Challenge from 2012 [19]. Our

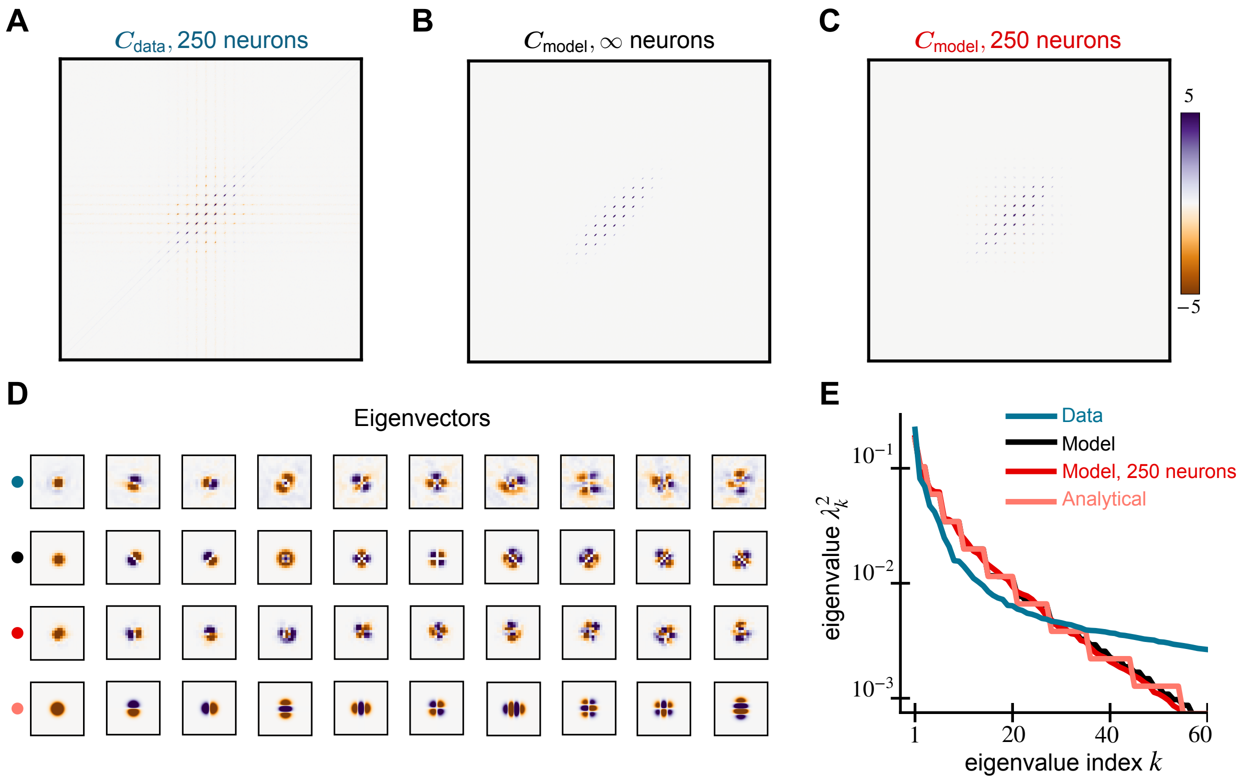


Figure K: **Spectral properties of V1 receptive fields and our model for Ringach dataset.** We compare the covariance matrices generated from the (A) receptive fields of 250 macaque V1 neurons, (B) the GP model Eq (21), and (C) 250 random samples from the model. The data is from [17]. (D) The leading 10 eigenvectors of the data and model covariance matrices show similar structure and explain 57% of the variance in the data. Analytical Hermite wavlet eigenfunctions are in the last row. (E) The eigenspectrum of the model matches well with the data.

implementation was based on the example provided by `pytorch` and `torchvision` [16] and used the same optimization routine, parameters, and schedule as in `https://github.com/pytorch/examples/tree/master/imagenet`.

All convolutional layers were initialized with weights drawn from a Gaussian distribution with variance $(c_{\text{in}} d_{\text{x}} d_{\text{y}})^{-1}$, where $c_{\text{in}}$ was the number of input channels, and $d_{\text{x}}$ and $d_{\text{y}}$ are the dimensions of the filter. This is equal to the reciprocal of the fan-in. In the case of classical initialization, this Gaussian distribution has covariance proportional to the identity, whereas in the structured case we use the V1-inspired covariance centered in the center of the filter with independent draws for each input channel. All biases and weights in the other layers are set with their `pytorch` defaults. The structured weights were only used in the first two convolutional layers of dimensions $d_{\text{x}} \times d_{\text{y}} = 11 \times 11$ and $5 \times 5$. The size parameter was set to $s = \max(d_{\text{x}}, d_{\text{y}}) \cdot 3$ and frequency bandwidth was $f = \max(d_{\text{x}}, d_{\text{y}})/5$.

We show training and testing loss over the first 10 epochs for both the classical and structured initializations in Fig. U. The structured initialization at first shows an advantage over classical, with consistently lower losses for the first 4 epochs, but eventually the classical network catches up. From this point onwards (until the 90 training epochs are complete), the classical network has the same or lower loss. Both networks end up performing well, reaching accuracies close to those reported in [18] and the `torchvision` documentation (`https://pytorch.org/vision/stable/models.html`), as shown in Table C. The classical initialization performs slightly better overall.

These null results are perhaps not surprising: The initial layers of AlexNet contain only 64 and 192 output channels (i.e. filters) respectively, making up only a small fraction of the total weights in the network. The deeper convolutional layers contain many more channels and are built with small 3×3 filters where our

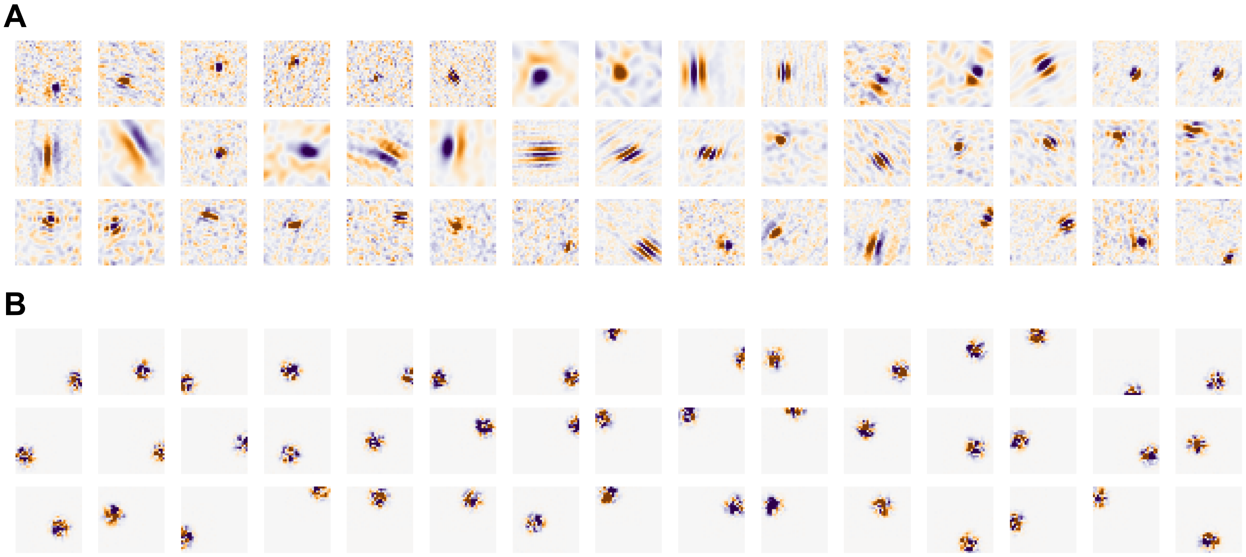


Figure L: **Receptive fields of V1 neurons from the Ringach dataset.** We show (A) biological receptive fields and (B) random samples from the fitted covariance model.

| Initialization | Loss | Top-1 accuracy (%) | Top-5 accuracy (%) |
|---|---|---|---|
| Classical | 2.059 (1.907) | 55.2 (56.5) | 76.3 (79.2) |
| Structured | 2.074 (1.920) | 53.0 (56.4) | 76.0 (79.0) |

Table C: **Results after training AlexNet for 90 epochs on ImageNet.** The classical initialization leads to slightly smaller loss and higher accuracy over the structured initialization. Test values are shown in parentheses; these are better than the training values due to dropout.

initialization is unlikely to help. It is also possible that the effects of initialization are less important for overparametrized models or with large amounts of training data.

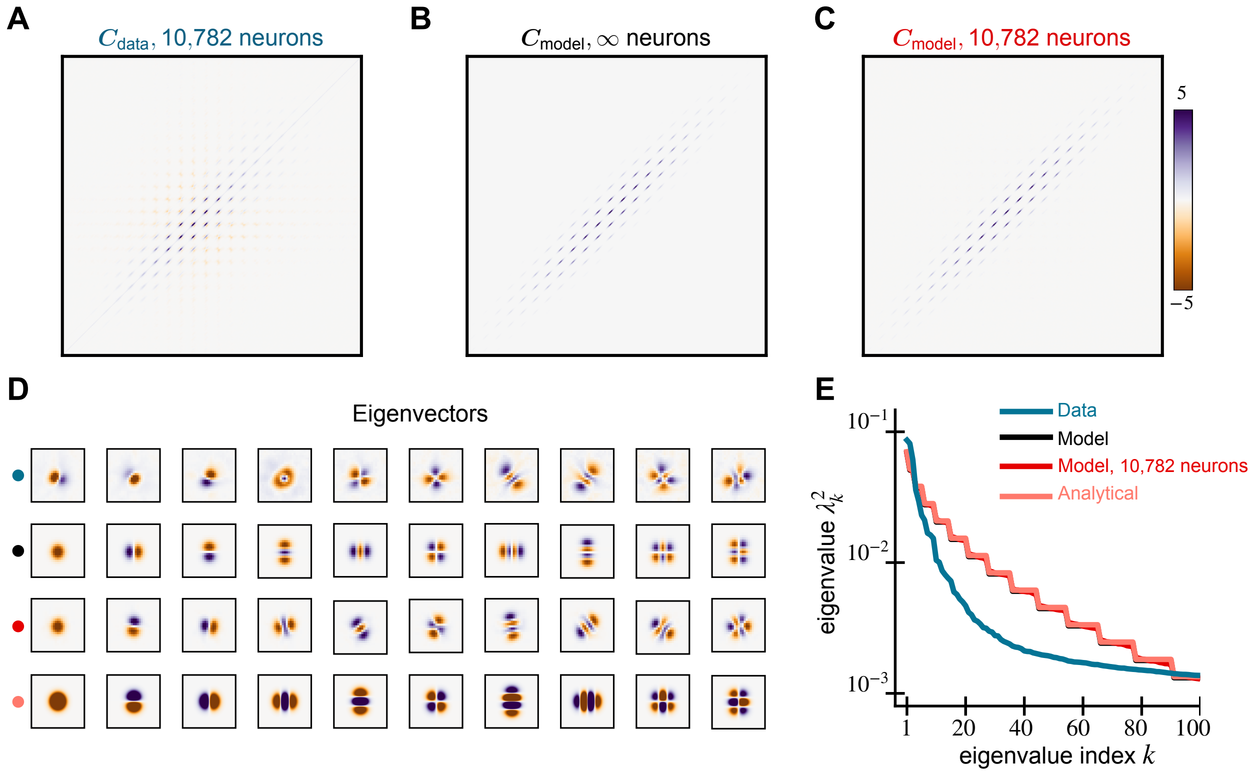


Figure M: **Spectral properties of V1 receptive fields and our model for natural image stimuli.** We compare the covariance matrices generated from the (A) receptive fields of 10,782 mice V1 neurons, (B) the GP model Eq (21), and (C) 10,782 random samples from the model. (D) The leading 10 eigenvectors of the data and model covariance matrices show similar structure and explain 39% of the variance in the data. Analytical Hermite wavelet eigenfunctions are in the last row. (E) The eigenspectrum of the model compared to the data.

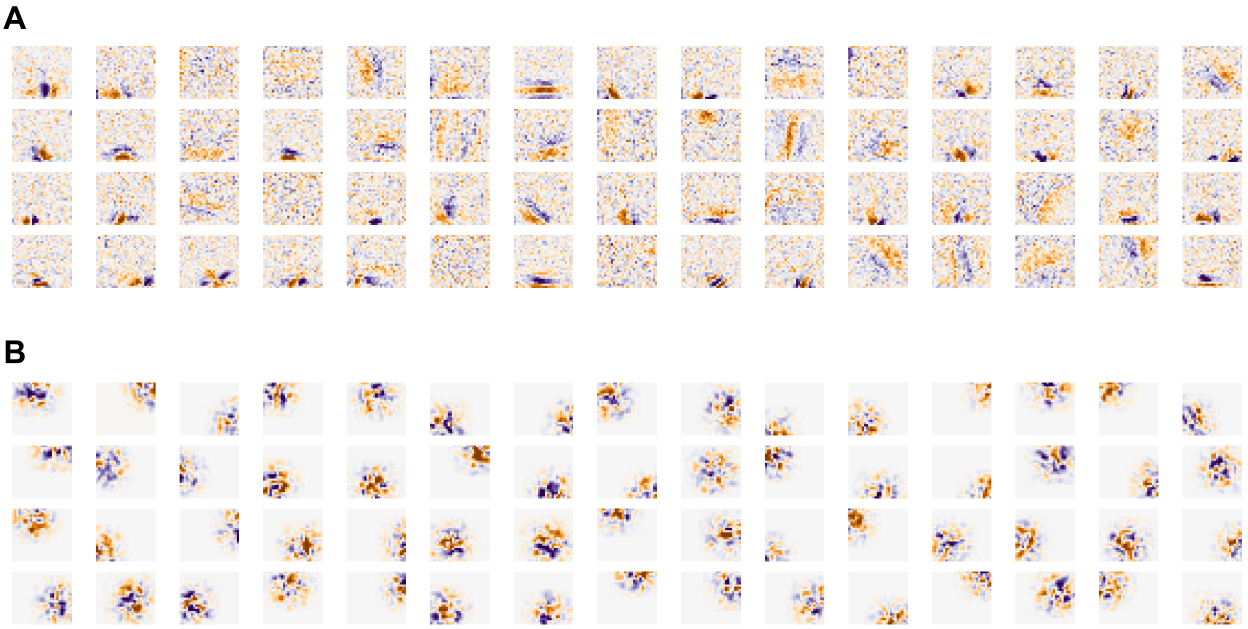


Figure N: **Receptive fields of V1 neurons from natural images stimuli.** We show (A) biological receptive fields and (B) random samples from the fitted covariance model.

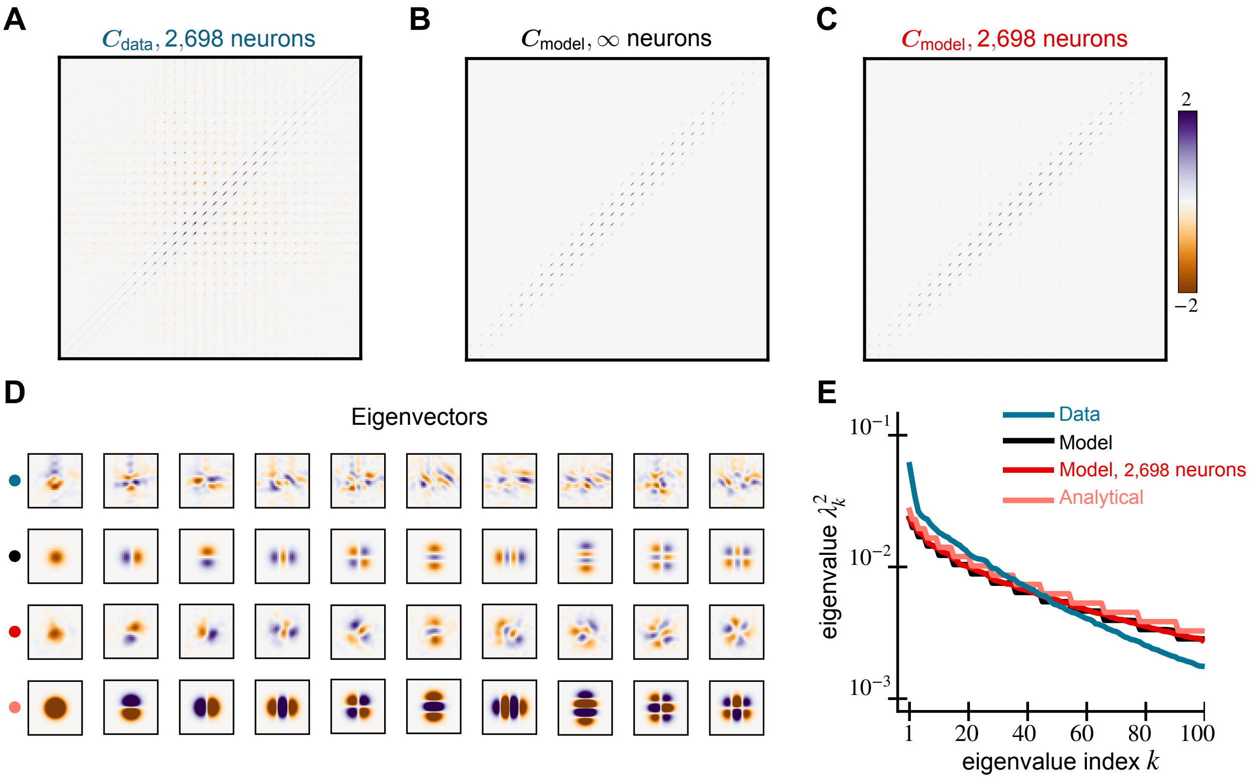


Figure O: **Spectral properties of V1 receptive fields and our model for DHT stimuli.** We compare the covariance matrices generated from the (A) receptive fields of 2,698 mice V1 neurons, (B) the GP model Eq (21), and (C) 2,698 random samples from the model. (D) The leading 10 eigenvectors of the data and model covariance matrices. They explain 29% of the variance in the data. Analytical Hermite wavelet eigenfunctions are in the last row. (E) The eigenspectrum of the model matches well with the data.

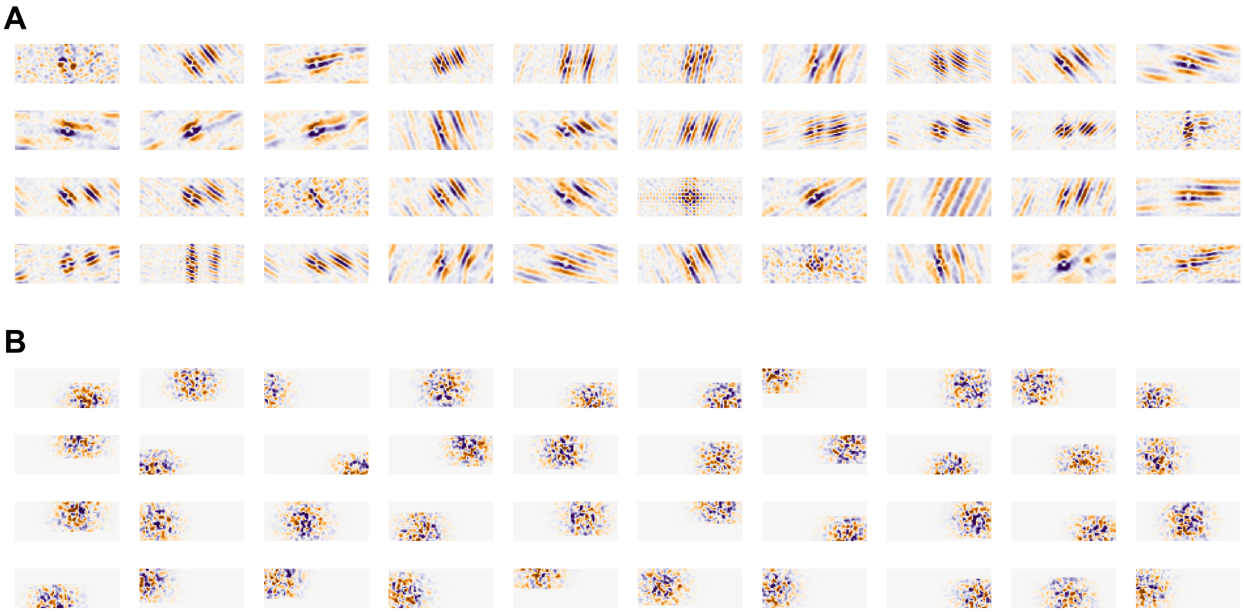


Figure P: **Receptive fields of V1 neurons from DHT stimuli.** We show (A) biological receptive fields and (B) random samples from the fitted covariance model.

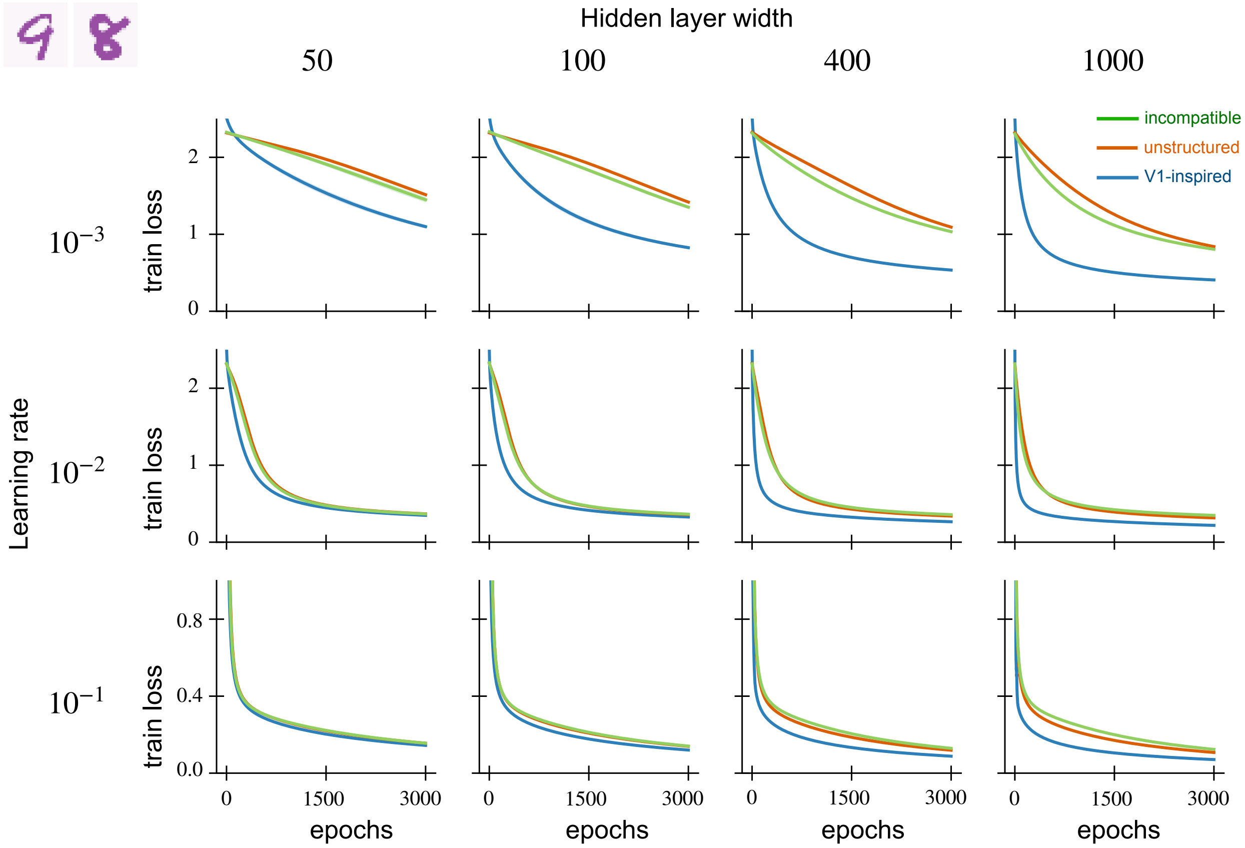


Figure Q: **Training loss on MNIST for fully-trained neural networks initialized with V1 weights.** We show the average training loss of fully-trained networks against the number of training epochs across diverse hidden layer widths (50, 100, 400, and 1000) and learning rates ($10^{-1}, 10^{-2}$, and $10^{-3}$). For every hidden layer width, we generate five random networks and average their performance. The solid lines show the average training loss while the shaded region represents the standard error. When the covariance parameters are tuned properly, V1-initialized networks achieve lower training loss over fewer epochs. The benefits are more significant at larger network widths and lower learning rates. With incompatible weights, V1 initialization leads to similar performance as unstructured initialization.

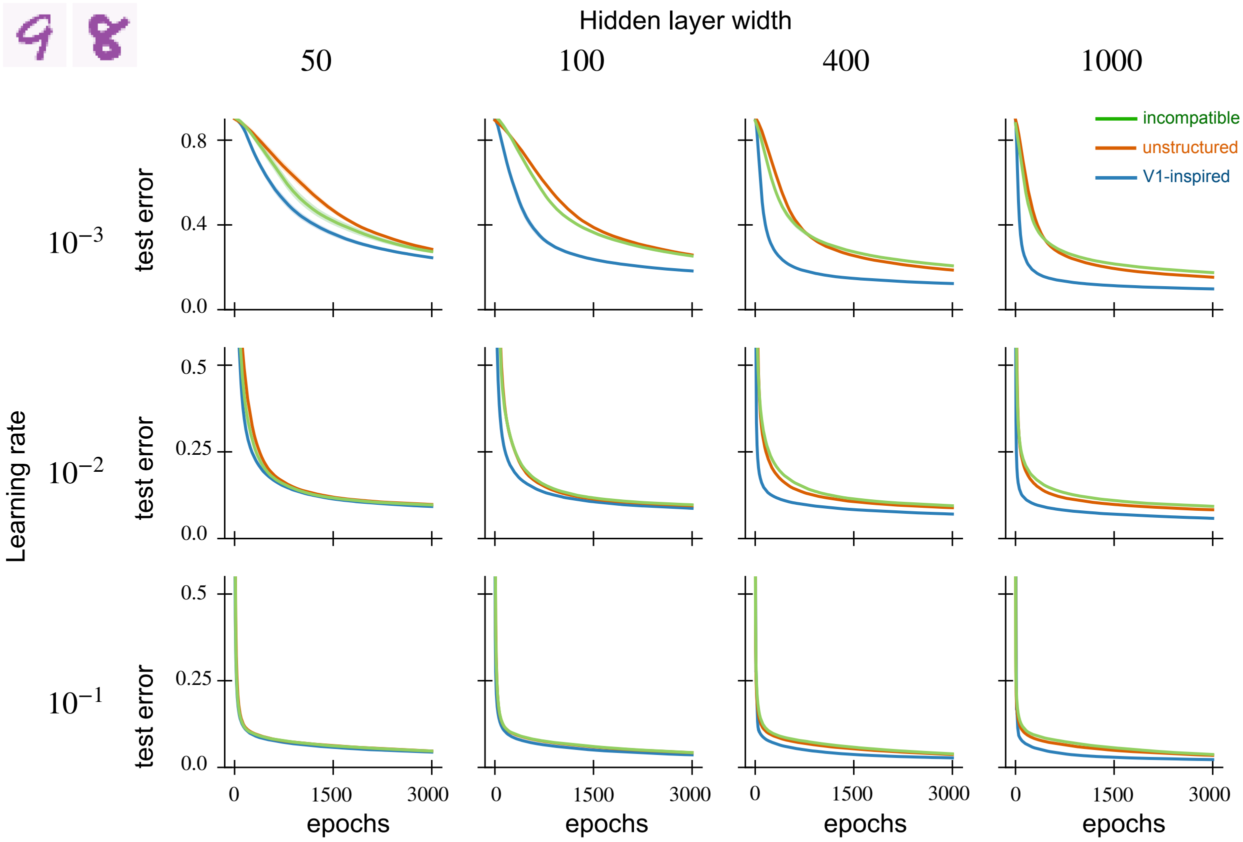


Figure R: **Test error on MNIST for fully-trained neural networks initialized with V1 weights.** We show the average test error of fully-trained networks against the number of training epochs across diverse hidden layer widths (50, 100, 400, and 1000) and learning rates ($10^{-1}, 10^{-2}$, and $10^{-3}$). For every hidden layer width, we generate five random networks and average their performance. The solid lines show the average test error while the shaded regions represent the standard error. When the covariance parameters are tuned properly, V1-initialized networks achieve lower test error over fewer epochs. The benefits are more significant at larger network widths and lower learning rates. With incompatible weights, V1 initialization leads to similar performance as unstructured initialization.

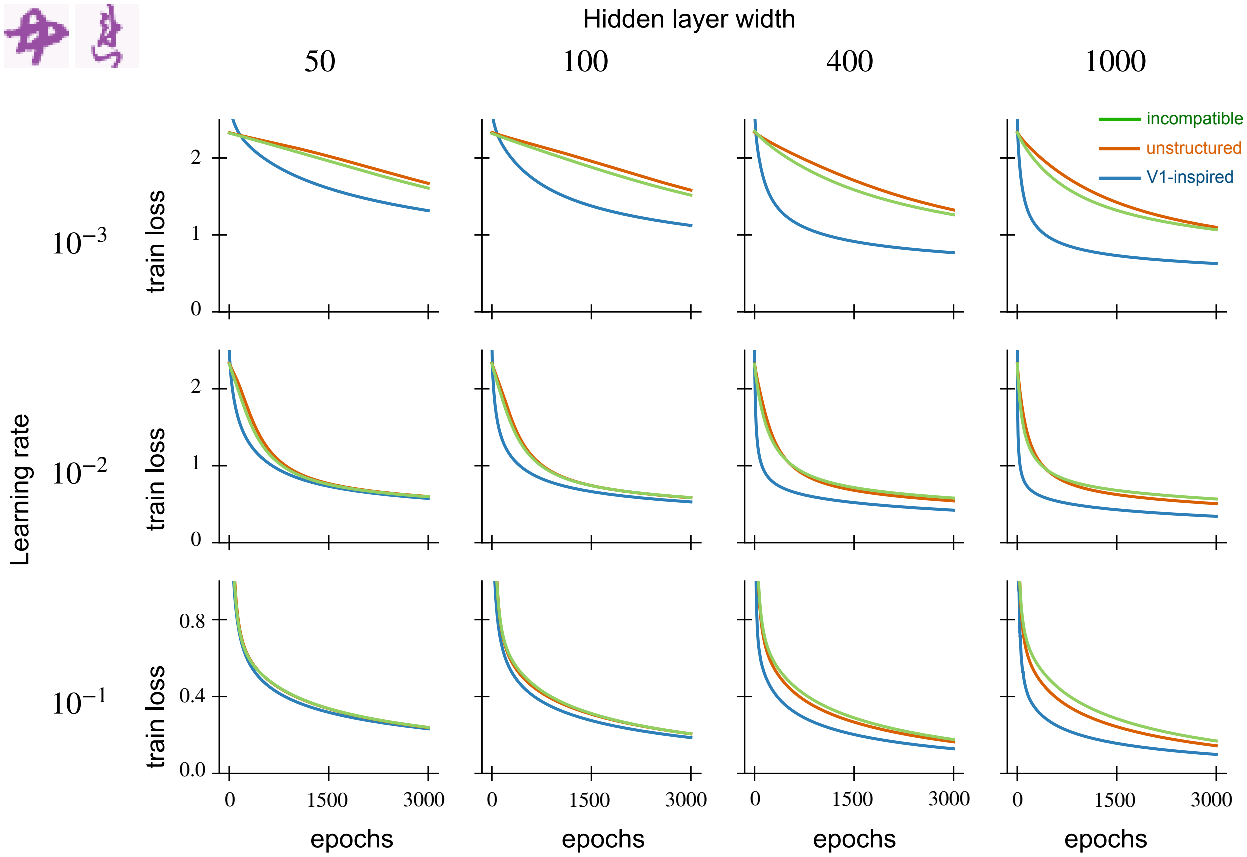


Figure S: **Training loss on KMNIST for fully-trained neural networks initialized with V1 weights.** We show the average training loss of fully-trained networks against the number of training epochs across diverse hidden layer widths (50, 100, 400, and 1000) and learning rates ($10^{-1}, 10^{-2}$, and $10^{-3}$). For every hidden layer width, we generate five random networks and average their performance. The solid lines show the average training loss while the shaded regions represent the standard error. When the covariance parameters are tuned properly, V1-initialized networks achieve lower training loss over fewer epochs. The benefits are more significant at larger network widths and lower learning rates. With incompatible weights, V1 initialization leads to similar performance as unstructured initialization.

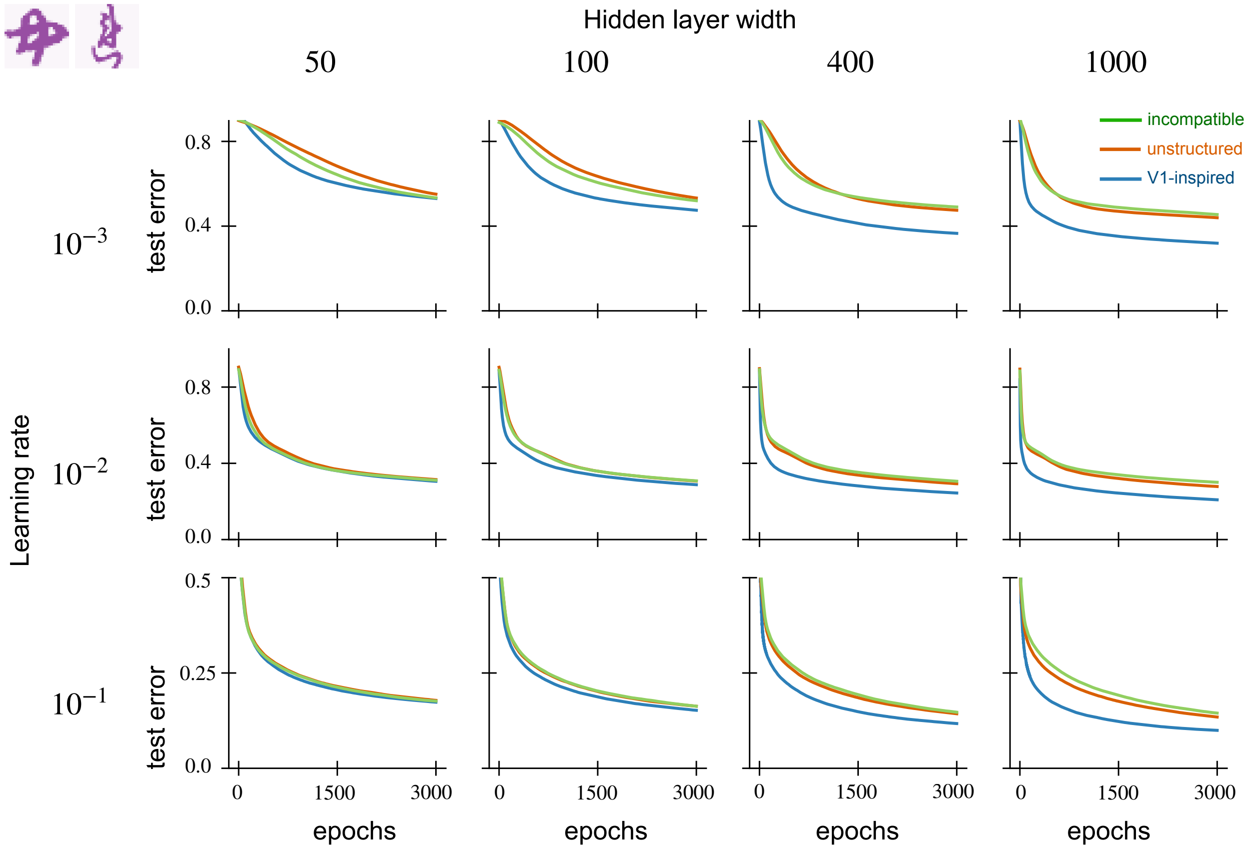


Figure T: **Test error on KMNIST for fully-trained neural networks initialized with V1 weights.** We show the average test error of fully-trained networks against the number of training epochs across diverse hidden layer widths (50, 100, 400, and 1000) and learning rates ($10^{-1}, 10^{-2}$, and $10^{-3}$). For every hidden layer width, we generate five random networks and average their performance. The solid lines show the average test error while the shaded regions represent the standard error. When the covariance parameters are tuned properly, V1-initialized networks achieve lower test error over fewer epochs. The benefits are more significant at larger network widths and lower learning rates. With incompatible weights, V1 initialization leads to similar performance as unstructured initialization.

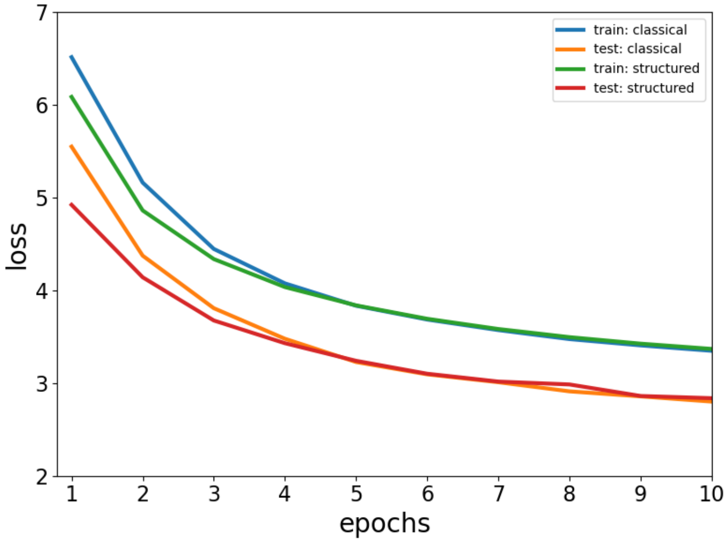


Figure U: **Initializing AlexNet using structured random features shows little benefit for ImageNet.** Training and testing loss are shown for classical and structured random initializations of convolutional layers in AlexNet. These losses are initially lower for structured features, but by 6 epochs the classical initialization catches up and it eventually reaches a slightly lower loss than the structured initialization. Note that the training losses are higher than testing due to dropout applied in the training phase.

# References


1. Cho Y, Saul L. Kernel Methods for Deep Learning. In: Bengio Y, Schuurmans D, Lafferty J, Williams C, Culotta A, editors. Advances in Neural Information Processing Systems. vol. 22. Curran Associates, Inc.; 2009. Available from: https://proceedings.neurips.cc/paper/2009/file/5751ec3e9a4feab575962e78e006250d-Paper.pdf.

2. Shawe-Taylor J, Cristianini N. Kernel Methods for Pattern Analysis. Cambridge University Press; 2004.

3. Shalev-Shwartz S, Ben-David S. Understanding Machine Learning: From Theory to Algorithms. Cambridge University Press; 2014.

4. Jacot A, Gabriel F, Hongler C. Neural Tangent Kernel: Convergence and Generalization in Neural Networks. In: Advances in Neural Information Processing Systems. vol. 31. Curran Associates, Inc.; 2018. Available from: https://papers.nips.cc/paper/2018/hash/5a4be1fa34e62bb8a6ec6b91d2462f5a-Abstract.html.

5. Canatar A, Bordelon B, Pehlevan C. Spectral Bias and Task-Model Alignment Explain Generalization in Kernel Regression and Infinitely Wide Neural Networks. arXiv:200613198 [cond-mat, stat]. 2021 Feb. ArXiv: 2006.13198. Available from: http://arxiv.org/abs/2006.13198.

6. Basri R, Galun M, Geifman A, Jacobs D, Kasten Y, Kritchman S. Frequency Bias in Neural Networks for Input of Non-Uniform Density. In: Proceedings of the 37th International Conference on Machine Learning. PMLR; 2020. p. 685-94. ISSN: 2640-3498. Available from: https://proceedings.mlr.press/v119/basri20a.html.

7. Abramowitz M, Stegun IA. Handbook of Mathematical Functions with Formulas, Graphs, and Mathematical Tables. U.S. Government Printing Office; 1964.

8. Strang G. The Discrete Cosine Transform. SIAM Review. 1999 Jan;41(1):135-47.

9. Bach FR. Exploring Large Feature Spaces with Hierarchical Multiple Kernel Learning. In: Koller D, Schuurmans D, Bengio Y, Bottou L, editors. Advances in Neural Information Processing Systems 21. Curran Associates, Inc.; 2009. p. 105-12.

10. Bach F. On the Equivalence Between Kernel Quadrature Rules and Random Feature Expansions. J Mach Learn Res. 2017 Jan;18(1):714-51.

11. Bordelon B, Canatar A, Pehlevan C. Spectrum Dependent Learning Curves in Kernel Regression and Wide Neural Networks. arXiv:200202561 [cs, stat]. 2020 Feb. ArXiv: 2002.02561. Available from: http://arxiv.org/abs/2002.02561.

12. Gradshteyn IS, Ryzhik IM. Table of integrals, series, and products. Seventh ed. Elsevier/Academic Press, Amsterdam; 2007.

13. Misiakiewicz T, Mei S. Learning with Convolution and Pooling Operations in Kernel Methods. arXiv; 2022.

14. Virtanen P, Gommers R, Oliphant TE, Haberland M, Reddy T, Cournapeau D, et al. SciPy 1.0: Fundamental Algorithms for Scientific Computing in Python. Nature Methods. 2020;17:261-72.

15. Harris CR, Millman KJ, van der Walt SJ, Gommers R, Virtanen P, Cournapeau D, et al. Array programming with NumPy. Nature. 2020;585:357–362.

16. Paszke A, Gross S, Massa F, Lerer A, Bradbury J, Chanan G, et al. PyTorch: An Imperative Style, High-Performance Deep Learning Library. In: Wallach H, Larochelle H, Beygelzimer A, d'Alché-Buc F, Fox E, Garnett R, editors. Advances in Neural Information Processing Systems 32. Curran Associates, Inc.; 2019. p. 8024-35. Available from: http://papers.neurips.cc/paper/9015-pytorch-an-imperative-style-high-performance-deep-learning-library.pdf.

17. Ringach DL. Spatial structure and symmetry of simple-cell receptive fields in macaque primary visual cortex. Journal of Neurophysiology. 2002 Jul;88(1):455–463.

18. Krizhevsky A. One weird trick for parallelizing convolutional neural networks. arXiv:14045997 [cs]. 2014 Apr. ArXiv: 1404.5997. Available from: http://arxiv.org/abs/1404.5997.

19. Russakovsky O, Deng J, Su H, Krause J, Satheesh S, Ma S, et al. ImageNet Large Scale Visual Recognition Challenge. International Journal of Computer Vision. 2015 Dec;115(3):211-52. Available from: https://doi.org/10.1007/s11263-015-0816-y.
