# Supplementary material for: Structured random receptive fields enable informative sensory encodings
Source: PLoS Comput Biol. 2022 Oct 10;18(10):e1010484. doi: 10.1371/journal.pcbi.1010484 (PMC9584455; doi:10.1371/journal.pcbi.1010484)
Supplement: Attachment — Submitted filename: cover_letter_resubmission.pdf [file pcbi.1010484.s002.pdf]

Dear Editors,

We are pleased to submit a revised version of our manuscript "**Structured random receptive fields enable informative sensory encodings**" for consideration at *PLOS Computational Biology.* We want to thank the reviewers for their detailed feedback on our work. Their feedback has given us new perspectives on our own work and certainly improved its quality. We have addressed all of the reviewers points one-by-one in the attached rebuttal.

Our resubmission is a major revision. On the exposition front, we have expanded the motivation behind our random receptive model and its connection to existing modeling methods. We describe the implication of our results on both neuroscience and deep learning more clearly. Based on the feedback, we have added a new appendix section on kernel theory with novel results. We mathematically demonstrate how structured random features improve learning for a concrete example.

To address the concerns with our experiments, we thoroughly cross-validated all the hyperparameters in our learning experiments. We also introduced several control models of receptive fields to compare with our existing GP covariance models and made minor improvements to both the text and the figures.

Below is a detailed list of our responses to the reviewer questions and the description of the manuscript changes. For the reviewer's convenience, we also show the exact changes we have made in the manuscript. Once again, we want to thank the reviewers for their time in engaging with our work and their feedback.

Sincerely,

Kameron Decker Harris

Reviewer 1:

1) Why is there a need for modeling receptive fields as random draws? This is not motivated at all. I found it strange that while the introduction of the paper reviews a wide range of literature (including even technical references to reproducing kernel Hilbert spaces whose relevance is questionable), the main motivation for this work is through a short sentence referring to data in ref. [8]. I went through that paper, and did not see how it motivates the authors' approach.

Thank you for a great question. Our motivation is primarily theoretical. By modeling receptive fields (RFs) as random draws, we can connect them to the theory of random features. This theory is one of the leading approaches we have of understanding how artificial neural networks (ANNs) learn from data. However, it hasn't seen much usage in the computational neuroscience literature. Our aim is to rectify this. As a proof of concept, we show that this framework can be fit to data and generate realistic RFs. From the framework, we can find explicit transformations used by neurons to transform the input data i.e. wavelet transformations in the space and time domain. All of this hinges on modeling receptive fields as random draws.

Our intention in citing the data in [8] was to show the discrepancy between the noisy but gabor-like RF data versus the clean Gabor filter models.

To address your point, we have emphasized our motivation more clearly in the abstract and introduction.

Abstract

"Brains must represent the outside world so that animals survive and thrive.
In early sensory systems, neural populations have diverse receptive fields structured to detect important features in inputs, yet significant variability has been ignored in classical models of sensory neurons. We model neuronal receptive fields as random, variable samples from parameterized distributions and demonstrate this model in two sensory modalities using data from insect mechanosensors and mammalian primary visual cortex. Our approach leads to a significant theoretical connection between the foundational concepts of receptive fields and random features, a leading theory for understanding artificial neural networks. The modeled neurons perform a randomized wavelet transform on inputs, which removes high frequency noise and boosts the signal. Further, these random feature neurons enable learning from fewer training samples and with smaller networks in artificial tasks. This structured random model of receptive fields provides a unifying, mathematically tractable framework to understand sensory encodings across both spatial and temporal domains."

Introduction
Given the stimulus presented and neural response data, one can then estimate the receptive fields of a population of neurons. Simple visual receptive fields have classically been understood as similar to wavelets with particular spatial frequency and angular selectivity \cite{Jones_Palmer_1987}. In mechanosensory areas, receptive fields are selective to temporal frequency over a short time window \cite{Fox_Fairhall_Daniel_2010}. Commonly, parametric modeling (Gabor wavelets \cite{stringer2019high}) or smoothing (regularization, etc. \cite{park2011receptive}) are used to produce ``clean'' receptive fields. Yet, the data alone show noisy receptive fields that are perhaps best modeled using a random distribution \cite{Bonin_2011}. As we will show, modeling receptive fields as random samples produces realistic receptive fields that reflect both the structure and noisiness seen in experimental data. More importantly, this perspective creates significant theoretical connections between foundational ideas from neuroscience and artificial intelligence. This connection helps us understand why receptive fields have the structures that they do and how this structure relates to the kinds of stimuli that are relevant to the animal.

2) What is the advantage of modeling receptive fields as random draws? While authors provide some evidence that their models capture second order statistics of experimental receptive fields, it is not clear what the advantage of this modeling approach is compared to, say, fitting an LN model to every neuron. The authors should state some performance metrics and make comparisons to other relevant techniques.

Our proposed method can fit an entire population of receptive fields using a handful of parameters and generate realistic receptive fields. In contrast, the LN model fits different parameters for individual receptive fields. This level of specificity does allow LN modeling to capture individual differences more accurately. Our models are simply not as flexible.

The advantage offered by our approach is the connection to the conceptual framework of random feature theory. Random features are one of the leading frameworks for theoretically understanding artificial (and, we argue, biological) neural networks. We have a modeling approach that not only generates realistic looking receptive fields but is also mathematically tractable. It allows us to concretely derive the mathematical transformations these receptive fields might use to transform their inputs for downstream processing, i.e. wavelet transforms in space and time. The direct connection to kernel learning opens the doorway to use techniques which are a mainstay in machine learning theory literature, like estimating generalization error and sample complexity, in the context of neuronal learning. While we haven’t explored those in this paper, new works like [Marjorie and Blake] have begun to use them.

To summarize, we do not claim that our proposed approach is better than existing modeling approaches like the LN model and should supplant them. If the goal is to capture individual differences between neuronal receptive fields, one should fit data with the LN model. Our perspective of modeling receptive fields as random draws opens up a whole new repertoire of

analysis techniques from machine learning theory that was unavailable before to computational neuroscience.

To address your point, we have added a paragraph in the Discussion (Section 3.5: Limitations and Future Directions).
The random feature receptive field model is a randomized extension of the LN neuron model. The LN model fits a parameterized function to each receptive field \cite{Chichilnisky_2001}. In contrast, the random feature framework fits a distribution to an entire population of receptive fields and generates realistic receptive fields from that distribution. A natural question is how they compare. If the goal is to capture individual differences between neuronal receptive fields, one should resort to an LN model where each neuron’s receptive field is fit to data. The random feature model is not as flexible, but it provides a direct connection to random feature theory, and it is mathematically tractable and generative. This connection to kernel learning opens the door to using techniques which are a mainstay in machine learning theory literature, for instance to estimate generalization error and sample complexity \cite{shawe-taylor_cristianini_2004}, in the context of learning in more biologically realistic networks.

3) Authors mention RKHSs a few times, and there is even some theoretical discussion in Appendix A.1. However, it is not clear how this connection is important or relevant. Reading the paragraph below equation 12 (sorry, no line numbers are provided), and discussion section 3.4, it feels like the authors want to claim that their learning efficiency results are related to learning in RKHSs. It would have been nice to see this point fully developed.

We mention RKHS in the Introduction, Discussion 3.4, and Appendix A.1 because of their connection to random feature networks. In particular, the function output by wide random feature networks lives in the RKHS. The geometry of this RKHS, by which we mean its inner product structure and eigenfunctions/eigenvalues, depends on the non-linearity and receptive field distribution of individual neurons. Results from kernel learning show us that if our target function has a small norm in this RKHS, it varies smoothly over the inputs. Smooth functions are easier to learn compared to fast varying ones. The point being that the choice of non-linearity and receptive field distribution affects the geometry and the ease of learning of our target function. We conjecture that receptive fields from neural-inspired distributions affect the RKHS geometry such that the target function’s norm is small in that RKHS, compared to the RKHS of random white-noise receptive fields.

To demonstrate this, we add a new Appendix section A.2 where we show how the RKHS geometry affects the norm of the target function for a simplified version of the frequency detection task. Given time series data, we define the target function that leads to perfect classification. We show that the norm of this function is larger in the RKHS of the structured features compared to that of unstructured features.

We do feel that it is beyond the scope of the paper to verify the above conjecture in detail for all of our tasks. Our goal in this work is to set up the connection of receptive fields to random feature neurons, emphasize the modeling work with real data, and show empirical results during learning. We leave the full theoretical verification of why we see learning efficiency for future work.

To address your point, we do two things:

1. **We have expanded upon the RKHS perspective in the Discussion (Section 3.4, Line 573).**
   An important open question for both neuroscience and machine learning is why certain networks, characterized by features such as their architecture, weights, and nonlinearities, are better than others for certain problems. One perspective is that a network is good for a problem if it is biased towards approximating functions that are close to the target, known as an {\em inductive bias}, which depends on an alignment between the features encoded by neurons and the task at hand \cite{Bordelon_2021}. Our approach shows that structured receptive fields are equivalent to a linear transformation of the input that can build in such biases. Furthermore, we can describe the nonlinear properties of the network using the kernel, which varies depending on the receptive field structure. If the target function has a small norm in this Reproducing Kernel Hilbert Space (RKHS), then there is an inductive bias and it is easier to learn \cite{shawe-taylor_cristianini_2004,Shalev-shwartz_ben-david_2014}.
   A small norm in the RKHS means that the target function varies smoothly over the inputs. Smooth functions are easier to learn compared to fast varying ones. In this way, the receptive field structure influences the ease of learning of the target function. We conjecture that receptive fields from neural-inspired distributions affect the RKHS geometry such that the target function's norm is small in that RKHS, compared to the RKHS of random white-noise receptive fields. We leave to future work to verify this conjecture in detail.

2. **We have added a new appendix section A.2 titled " Kernel theory for frequency detection: a fully worked, highly nutritious, simplified example."** There we demonstrate how this change of basis leads to a smaller norm of the target norm for a simplified version of the frequency detection task.

4) Instead of MNIST, authors could use a dataset with more naturalistic and complex stimuli.

We agree with your point and for this reason used KMNIST (a more difficult task of classifying Japanese characters) and the natural image dataset Imagenet. In the last paragraph of Section 2.3.5, we mention our results from classifying ImageNet by initializing the early convolutional layer of AlexNet with our random V1 receptive fields. We did not see any benefits to training when compared to standard initialization techniques. We provide the excerpt from the text below.

“However, the improvement is not universal: no significant benefit was found by initializing the early convolutional layers of the deep network AlexNet [48] and applying it to the ImageNet dataset [75], as shown in Appendix A.10. The large amounts of training data and the fact that only a small fraction of the network was initialized with structured weights could explain this null result.”

5) A theoretical study of what makes a receptive field covariance compatible or incompatible with downstream learning should be pursued. This may be related to the RKHS picture that the authors are hinting at.

Thank you for your idea! This is a very interesting theoretical direction to pursue. We agree with you that the answer to this question is related to the RKHS picture. The covariance determines the associated RKHS kernel. The RKHS norm of the target function (required for downstream learning) determines its smoothness (with respect to the metric defined by the associated RKHS kernel). The smoother a function is, the easier it is to learn. Thus, receptive fields whose covariance structure leads to a smaller norm are more compatible with downstream learning. In the kernel learning literature, it is well-known that choosing or constructing a good kernel for the task makes all the difference in learning.

However, we believe that the question lies beyond the scope of this paper. There are several theoretical works whose focus was to answer this question. For instance, Borderlon et. al. showed that the covariance structure of V1 receptive fields enables them to be good at tasks that depend on low spatial frequency components. Harris et. al. showed that the covariance structure of associative centers like the cerebellum allows them to behave like additive kernels. Such kernels are really good at learning in high dimensions in the presence of random noise and adversarial examples. We outline the full excerpt below from our paragraph 6 in the introduction that describes these results in detail.

“Bordelon, Canatar, and Pehlevan, in a series of papers, have shown that neural codes allow learning from few examples when spectral properties of their second-order statistics aligns with the spectral properties of the task [9, 10, 13]. When applied to V1, they found that the neural code is aligned with tasks that depend on low spatial frequency components. Harris constructed

an RFN model of sparse networks found in associative centers like the cerebellum and insect mushroom body and showed that these areas may behave like additive kernels [34], an architecture also considered by Hashemi et al. [35]. These classes of kernels are beneficial for learning in high dimensions because they can learn from fewer examples and remain resilient to input noise or adversarial perturbation. Xie et al. investigated the relationship between the fraction of active neurons in a model of the cerebellum—controlled by neuron thresholds—and generalization performance for learning movement trajectories [89].
“

We hope that our change for comment #3 addresses this point. There, we expanded upon the RKHS perspective in the Discussion (Section 3.4 Para 1) and added a new section A.2 titled “ Kernel theory for frequency detection: a fully worked, highly nutritious, simplified example.”.
“An important open question for both neuroscience and machine learning is why certain networks, characterized by features such as their architecture, weights, and nonlinearities, are better than others for certain problems. One perspective is that a network is good for a problem if it is biased towards approximating functions that are close to the target, known as an {\em inductive bias}, which depends on an alignment between the features encoded by neurons and the task at hand \cite{Bordelon_2021}. Our approach shows that structured receptive fields are equivalent to a linear transformation of the input that can build in such biases. Furthermore, we can describe the nonlinear properties of the network using the kernel, which varies depending on the receptive field structure. If the target function has a small norm in this Reproducing Kernel Hilbert Space (RKHS), then there is an inductive bias and it is easier to learn \cite{shawe-taylor_cristianini_2004,Shalev-shwartz_ben-david_2014}.
A small norm in the RKHS means that the target function varies smoothly over the inputs. Smooth functions are easier to learn compared to fast varying ones. In this way, the receptive field structure influences the ease of learning of the target function. We conjecture that receptive fields from neural-inspired distributions affect the RKHS geometry such that the target function’s norm is small in that RKHS, compared to the RKHS of random white-noise receptive fields. We leave to future work to verify this conjecture in detail.”

6) The authors have a great list of possible future directions. One or more of these directions could be pursued for this paper.
We definitely agree with you. We expand upon the RKHS perspective for this paper and leave the further directions for follow up works.

Other
1) Black line in Figure 5D is not visible.
We believe you are referring to the black curve in Figure 5E. We’ve added a statement to the caption: “The model curve with infinite neurons (black) is obscured by the model curve with 8,358 neurons (red).” Thank you for catching this.

Reviewer 2:

1. The results on the matching to biological receptive fields are based on qualitative--not quantitative--comparisons. The authors compare the covariance matrices from experimental data with those fitted by the model only based on how similar they look to each other on a plot. One way in which the results might be strengthened is by including a null hypothesis, like purely random receptive fields, or receptive fields based on Gabor filters for V1.

Thank you for your suggestion. The way we match the model to data is that we construct a cost function over the covariance model parameters that measures the Frobenius norm of the difference between the model covariance matrix and the data covariance matrix. We minimize this objective to find the best fitting parameters. The resulting model covariance we show in Fig 3A and Fig 5A are the best quantitative fits to the data. Our results on matching to biological receptive fields are quantitative in that sense. The objective function is shown below and is in the Appendix A.2.1 for mechanosensor data and A.2.2 for V1 data:

(sensilla data) $f_{lo}, f_{hi,} \gamma := \min_{f_{lo,} f_{hi,} \gamma} || C_{model}(f_{lo,} f_{hi}, \gamma) - C_{data}||_F$ subject to $f_{hi} \geq f_{lo}$

(V1 data) $s_{min}, f_{min} := \min_{s, f} || C_{model}(s, f) - C_{data}||_F$

In the text, we relegate these details in the appendix section and perhaps emphasize the qualitative match a lot more.

To address your comment, we do several things:

- **We emphasize this minimization procedure over the Frobenius norm in Section 2.2.3 Paragraph 8. We also mention the value of the normalized Frobenius norm difference between the data and the model in the text.**
  "We again optimize the parameters to fit the data (Appendix~\ref{app:cov_opt_V1}), finding $s=1.87$ and $f=0.70$ pixels. We do so by minimizing the Frobenius norm of the difference between $\mat C_{\rm{data}}$ and the model. We do not need to optimize over the center parameter $\vec{c}$, since we preprocess the data so that all receptive fields are centered at $\vec{c} = (7, 18)$, the center of the 14$\times$36 grid. The resulting model covariance matrix (Fig.~\ref{fig:cov_V1}B) and the data covariance matrix (Fig.~\ref{fig:cov_V1}A) match remarkably well qualitatively. **The normalized Frobenius norm of the difference between $\mat C_{\rm{data}}$ and the model is $0.2993.$"**

- **We added a test against a null hypothesis. We show covariance matrices from two null V1 models: purely random receptive fields and translation invariant models of**

the existing RF model and mention their normalized Frobenius norm difference. In the translation invariant model, we remove the localization term from the covariance function in Equation 9. Now, the receptive field covers the entire visual field. We still fit the spatial frequency parameter. The equation for the covariance function is below.

(Full model) $C(t,\ t') \ = \ exp(\frac{-|t-t'|^2}{2\,f^2}) \cdot exp(\frac{-|t-c|^2 + -|t'-c|^2}{2\,s^2})$

(Translation invariant model) $C(t,\ t') \ = \ exp(\frac{-|t-t'|^2}{2\,f^2})$

(Purely Random model) $C(t,\ t') \ = I$

We add the following null model comparison paragraph in Section 2.2.3 (Paragraph 9). We show the covariance matrices of these V1 null models in Appendix A.4. We also show the new figures below.

"For comparison, we also calculate the Frobenius difference for our null models, the unstructured covariance model and the translation invariant V1 model (\ref{eq:cov_V1_trans_inv}). In the translation invariant model, we remove the spatially localizing exponential in Eq. \ref{eq:cov_V1} and only fit the spatial frequency parameter, f. **For the purely random receptive fields, the Frobenius norm difference is 0.9835 while that of the translation invariant model model is 0.9727.** Thus, V1 covariance model fits the data more accurately compared to the null hypotheses. We show the covariance matrices from these null models in the Appendix \ref{app:null_models_v1}."

The covariance matrices for the null receptive field models of V1.

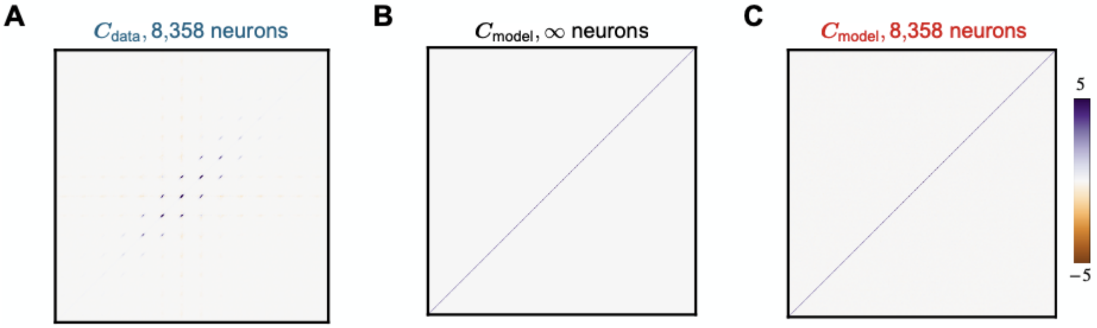

Figure 9: **Covariance matrix of V1 receptive fields and unstructured model for white noise stimuli.** We compare the covariance matrices generated from the (A) receptive fields of 8,358 mice V1 neurons, (B) unstructured GP model and (C) 8,358 random samples from the model.

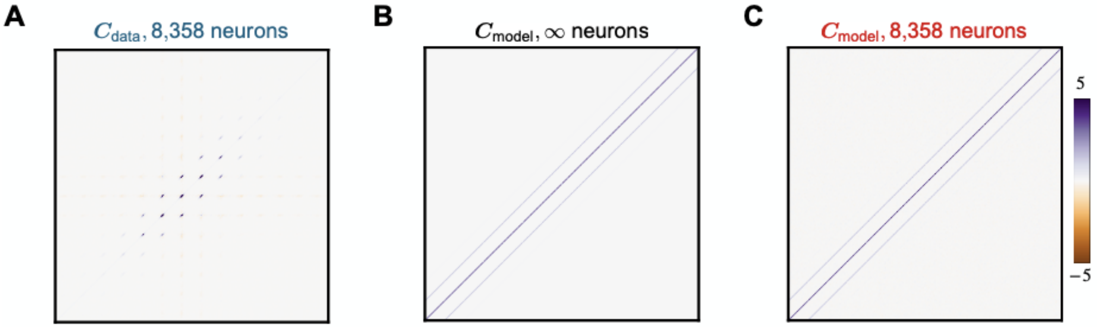

Figure 10: **Covariance matrix of V1 receptive fields and translation invariant V1 model (Eq. 15) for white noise stimuli.** We compare the covariance matrices generated from the (A) receptive fields of 8,358 mice V1 neurons, (B) translation invariant version of the GP model and (C) 8,358 random samples from the model.

- **We emphasize this minimization procedure over the Frobenius norm also in Section 2.2.3 Paragraph 8 for the mechanosensory neurons. We also mention the value of the normalized Frobenius norm difference between the data and the mechanosensory model in the text.**
  "To fit the covariance model to the data, we optimize the parameters (see Appendix~\ref{app:cov_opt}) $f_\mathrm{lo}, f_\mathrm{hi}$, and γ, finding $f_\mathrm{lo}=75$ Hz, $f_\mathrm{hi}=200$ Hz, and $\gamma = 12.17$ ms best fit the sensilla data. We do so by minimizing the Frobenius norm of the difference between $\mat C_{\rm{data}}$ and the model (\ref{eq:opt_mech}). The resulting model covariance matrix (Fig.~\ref{fig:cov_mech}B) matches the data covariance matrix (Fig.~\ref{fig:cov_mech}A) remarkably well qualitatively. **The normalized Frobenius norm of the difference between $\mat C_{\rm{data}}$ and the model is $0.4386.$"**

- **We compare with null models for the mechanosensory neurons, namely the fourier model we mention in Section 2.2.1 and the unstructured model. We add the following null model comparison paragraph in Section 2.2.2 (Paragraph 8). We show the covariance matrices of these sensilla null models in Appendix A.4. We also show the new figures below.**
  "For comparison, we also calculate the Frobenius difference for null models, the unstructured covariance model and the fourier model (\ref{eq:cov_fourier}). **For the unstructured model, the Frobenius norm difference is 0.9986 while that of the fourier model is 0.9123.** The sensilla covariance model has a much lower difference compared to the null models and thus, fits the data more accurately. We show the covariance matrices from these null models in the Appendix \ref{app:null_models_mech}."

The covariance matrices for the null receptive field models of mechanosensory neurons.

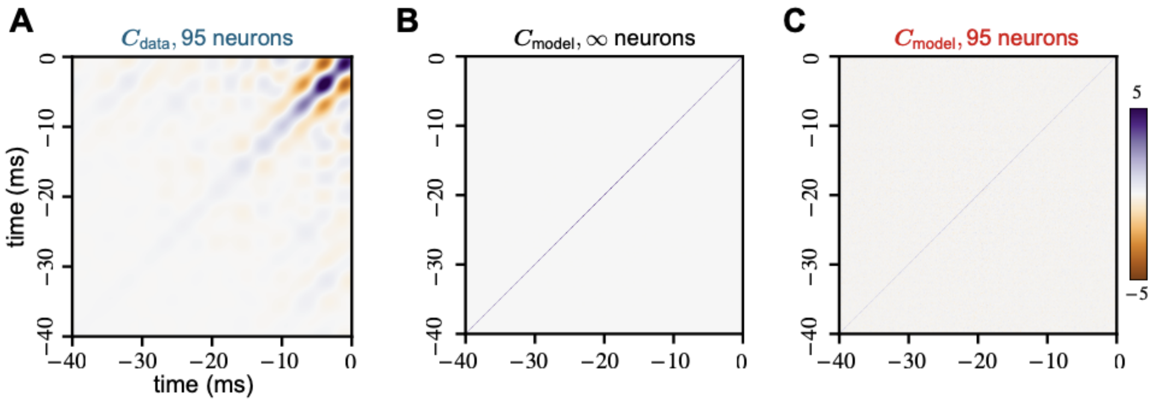

Figure 9: **Covariance matrix of mechanosensory receptive fields and unstructured model.** We compare the covariance matrices generated from the (A) receptive fields of 95 mechanosensory neurons, (B) unstructured GP model and (C) 95 random samples from the model.

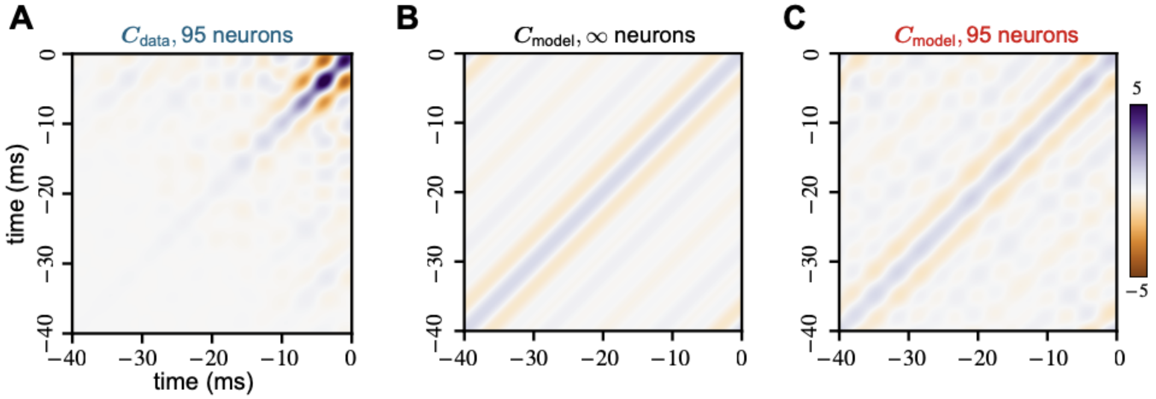

Figure 10: **Covariance matrix of mechanosensory receptive fields and the fourier model (5).** We compare the covariance matrices generated from the (A) receptive fields of 95 mechanosensory neurons, (B) fourier GP model and (C) 95 random samples from the model.

2. The authors do a nice job of motivating their theory with the theory of random feature networks from the machine learning literature. I would also have liked to see the authors discuss in further detail their ideas in the context of previous computational neuroscience approaches to describing receptive fields, including efficient coding, sparse coding, and related approaches.

Thank you for your suggestion. It is not obvious to us the direct consequences of our results for these other theories. It does seem likely that wavelet ideas, core to sparse coding principles at least in vision, provide a solid connection. We have the following to the discussion to make clear that these other approaches can offer a complementary perspective:

To address your point, we add a short section to Discussion Section 3.1.
"The random feature formulation is a natural extension of the traditional linear-nonlinear (LN) neuron model. This approach may be applied to other brain regions where LN models are successful, for instance sensory areas with primarily feedforward connectivity like somatosensory and auditory regions. The neurons in auditory and somatosensory systems are selective to both spatial and temporal structures in their stimuli \cite{Knudsen_1978, Hsiao_2008,Pruszynski_2014}, and spatial structure emerges in networks trained on artificial tactile tasks \cite{Zhao_2018}. Their receptive fields could be modeled by GPs with spatiotemporal covariance functions \cite{Xu_2021}; these could be useful for artificial tasks with spatiotemporal stimuli such as movies and multivariate timeseries. Neurons with localized but random temporal responses were found to be compatible with manifold coding in a decision-making task \cite{Koay_2021}. Our GPs are a complementary approach to traditional sparse coding \cite{olshausen1997} and efficient coding \cite{barlow1961,chalk2018} hypotheses; the connections to these other theories are interesting for future research."

3. For establishing that structured weights lead to faster learning in Section 2.3.5, a more convincing way to show this in our view would be to (i) define a metric for speed of learning (e.g. area under the learning curve) (ii) optimize the learning rate separately for each network with respect to this metric, and (iii) compare this metric for the two networks with their separately optimized learning rates. The way that the simulations are currently being done leaves open the possibility that the slower learning in the unstructured case might be due to a suboptimal choice of learning rate.

This is a great suggestion! We didn't think of defining such a metric and optimizing the learning rate for each network as the area under the learning curve. We picked the learning rates using grid search over the range of [1e-3, 1e0] and picked the one that minimizes the training loss the most at the end of 3000 epochs.

However, we liked this suggestion. We implemented it by performing a grid search for the learning rate that minimizes the area under the learning curve metric in the range of [1e-4, 1e0] divided into 20 intervals logarithmically. Any higher value of learning rate brings up convergence

issues. The optimal learning rates we get are 0.23357215, 0.14384499, 0.14384499 for structured, unstructured, and incompatible networks, respectively. We show the learning curves from this optimal value of the learning rate for each network. The results from the simulations didn't change from before and look virtually the same qualitatively. The trend continues to hold. The absolute values of the loss and test error did decrease somewhat across all networks as a result of the learning rate optimization.

To address your comment, we performed the simulation and made the following change the Section 2.3.5 paragraph 3. We also updated Figure 8.
"We again compare structured and unstructured weights on MNIST and KMNIST tasks, common benchmarks for fully-trained networks. The architecture is a single hidden layer feedforward neural network (Fig.~\ref{fig:network_architecture}) with 1,000 hidden neurons. The cross-entropy loss over the training sets are minimized using simple gradient descent (GD) for 3,000 epochs. For a fair comparison, the learning rate is optimized for each network separately. We define the area under the training loss curve as a metric for the speed of learning. Then, we perform a grid search in the range of $(1e^{-4}, 1e^{0})$ for the learning rate that minimizes this metric, resulting in the parameters $0.23, 0.14, 0.14$ for structured, unstructured and incompatible networks respectively. All other parameters are the same as in Section \ref{sec:image_clf}."

The old Figure 8 is below.

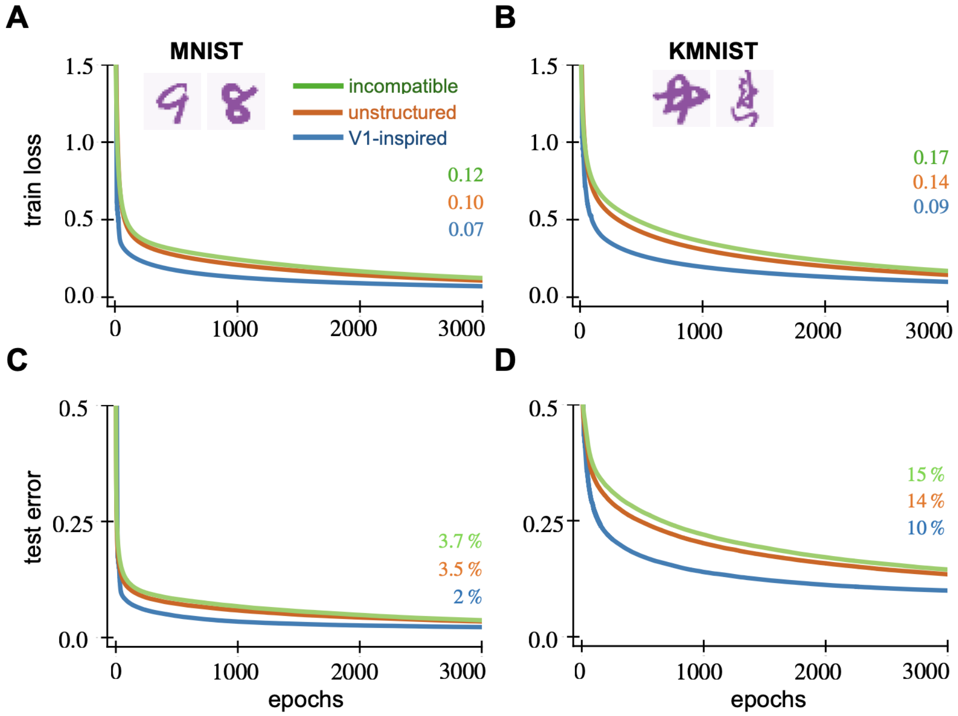

The updated Figure 8 is below.

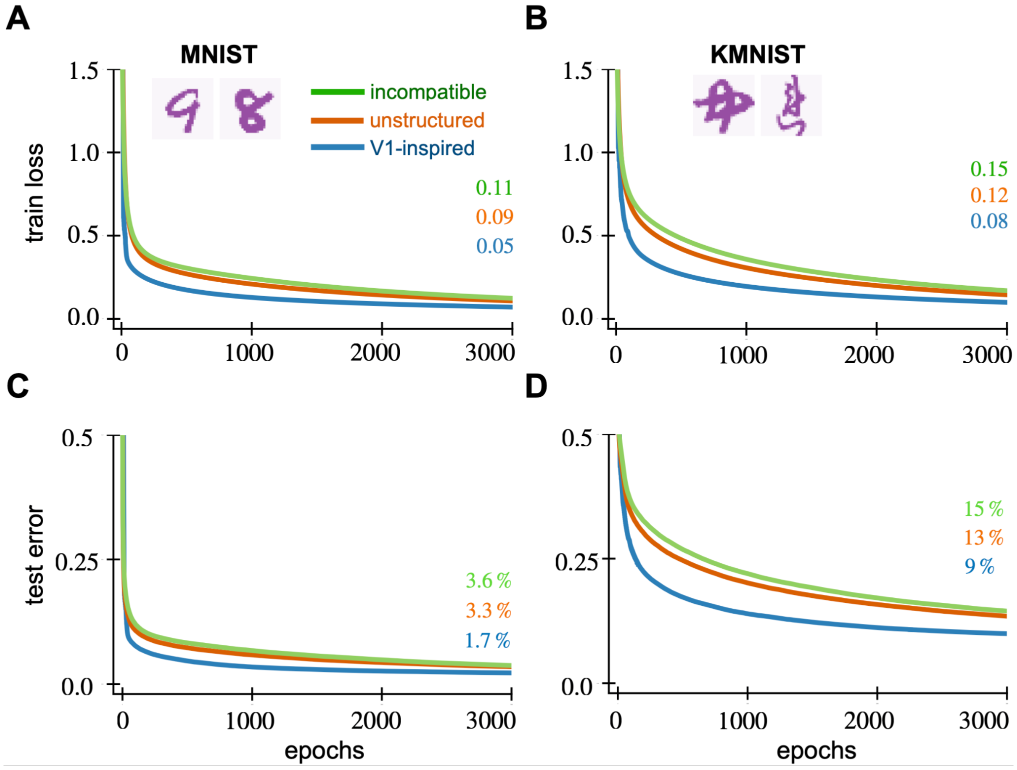

Minor points

4. In section 2.1.1, can the authors explain why the L2 regularization parameter was set to 1? Is it possible that any of the presented results would change if this parameter were set differently?

Thank you for the question. We agree it is possible that the results could change with hyperparameter tuning, so we tuned the L2 regularization parameter according to your suggestion.

For each task and each network size, we performed a grid search for the L2 regularization parameters and found the best parameter via 5-fold CV. Our search space was over the range of [1e-3, 1e3] divided into 6 intervals. When we pick the best parameter for each network size, we find that the classification error rate decreases across all networks but the trends hold. The neural inspired networks still outperform the unstructured networks. The neural-inspired networks with incompatible parameters, i.e. incorrect inductive bias, still perform worse than unstructured networks.

To address your point, we did several things.

1. **We added the following clarification in Section 2.1.1**
   "In our RFN experiments, we train the readout weights $\vec \beta \in \R^{m}$ and offset $\beta_0 \in \R$ using a support vector machine (SVM) classifier with squared hinge loss and ℓ^2 penalty with regularization strength of tuned in the range $[10^{-3}, 10^3]$ by 5-fold cross-validation. Our RFNs do not include a threshold for the hidden neurons."

2. **We updated Figures 6 and 7 after tuning the L2 regularization parameter.**

Below is the original Figure 6 below with the L2 value equal to 1.0. These plots are for the frequency detection task.

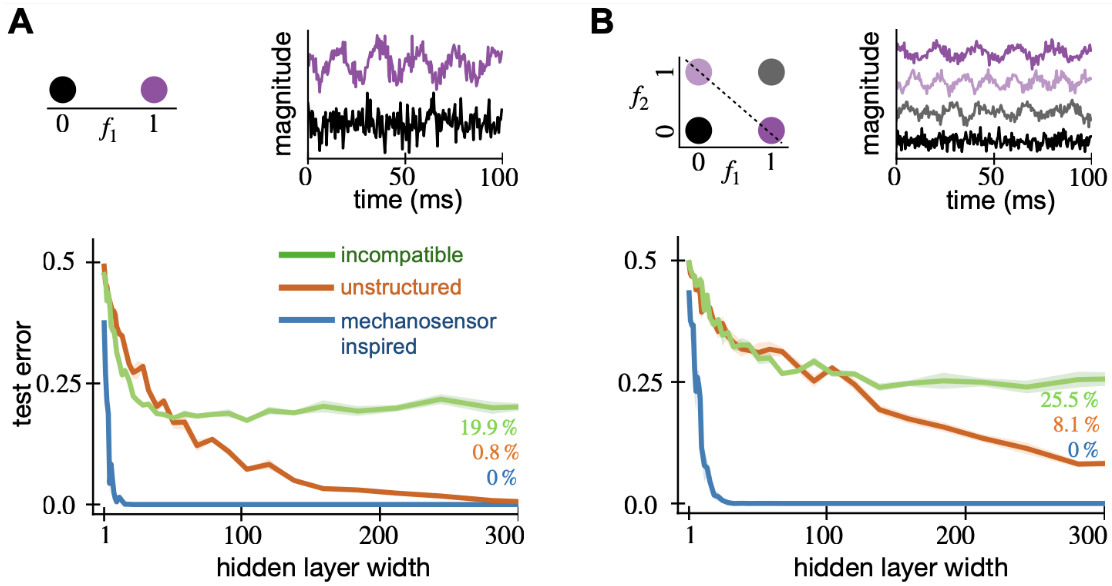

Here, we show the classification error after tuning the regularization parameter for each network size. We see that optimizing over the L2 penalty does improve the classification error but the effect is over all models. The trend still holds.

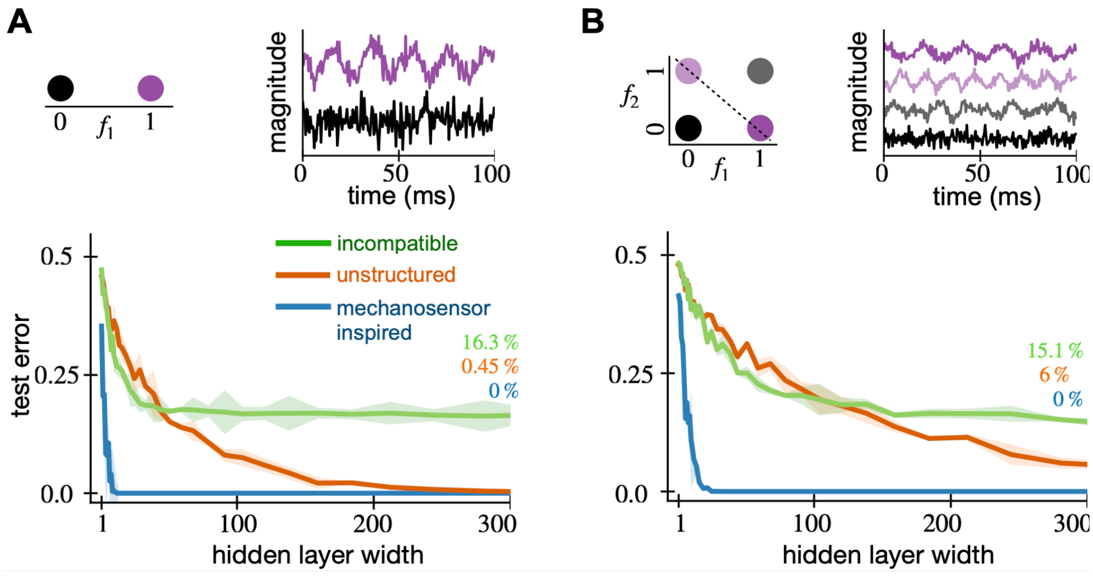

Below is the original Figure 7 below with the L2 value equal to 1.0. These plots are for the digit classification task.

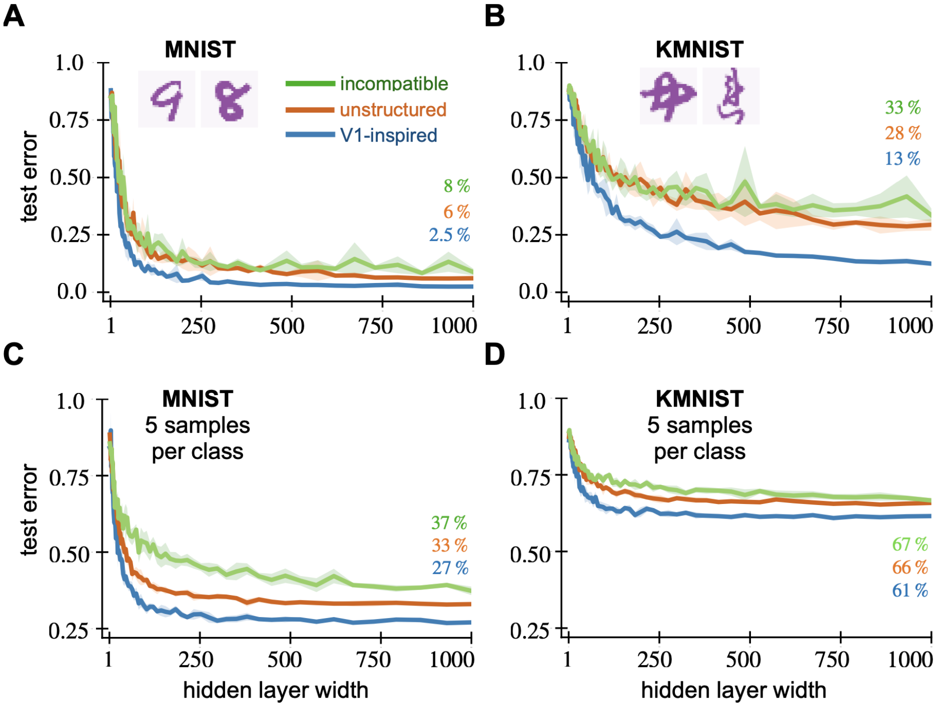

Here, we show the classification error after tuning the regularization parameter for each network size. We get identical classification errors for the largest network size here. The trend still holds.

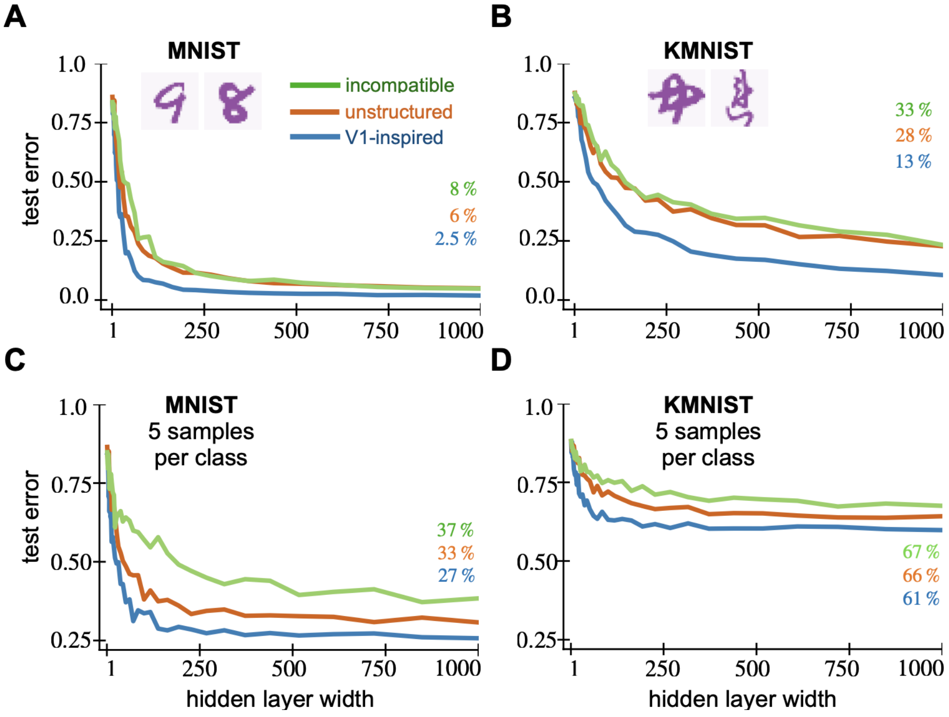

5. In the first paragraph of Section 2.1.3, the claim is made, without specifying any particular task, that adding an inductive bias improves learning. The authors should be careful about statements like this here and elsewhere in the paper since, as they are obviously aware given that they illustrate this point themselves in the simulations, an inductive bias can only be expected to improve learning if it is well matched to the particular computational task, and it can actually worsen learning if this is not the case.

This an excellent point. You're right that inductive bias only makes sense with a particular task at hand. We made sure to clarify that we are working with tasks with naturalistic inputs when we talk about the inductive bias from neuronal receptive fields improving learning.

We do the following changes to address your point:

1. **We made this addition for Section 2.1.3, Line 170.**
   "In Section~\ref{sec:experiments}, we will see that this adds an inductive bias to classical RFNs for the classification tasks with naturalistic inputs and improves learning."

2. **We made this addition in Section 2.3, Line 366.**
   "Our hypothesis is that neuronal inductive bias from structured receptive fields allows networks to learn with fewer neurons, training examples, and steps of gradient descent for classification tasks with naturalistic inputs.

6. In Section 2.1.3, the authors state "the GP covariance function reflects the statistical regularities within the sensory inputs to the network". Can the authors provide a citation for this statement in the cases considered?
Thank you for this question. We decided to remove this sentence from the paragraph.

"We generate network weights from Gaussian processes (GP) whose covariance functions are inspired by the receptive fields of sensory neurons in the brain.
By definition, a GP is a stochastic process where finite observations follow a Gaussian distribution \cite{Rasmussen_2005}. ~~We design the weight covariance of the GP so that our weights are compatible with our inputs, which is to say, the GP covariance function reflects the statistical regularities within the sensory inputs to the network.~~ We find that networks with such weights project inputs into a new basis and filter out irrelevant components. In Section~\ref{sec:experiments}, we will see that this adds an inductive bias to classical RFNs for tasks with naturalistic inputs and improves learning."

7. In the second-to-last paragraph of section 2.2.1, the bandwidth should be f_hi - f_lo.
Thank you for catching this. We made this correction.

8. In equation (7) of section 2.2.1, why are the stochastic coefficients for cos(\omega_k t) and sin(\omega_k t) not independent?
Thank you for catching this. The coefficients are indeed independent and we have fixed it in the text in Section 2.2.1, Equation 7.

9. At the end of section 2.2.1, can the authors more precisely explain the statement 'The smoothness is also controlled by the overall magnitude of the nonzero eigenvalues'?
This is a good observation. We were vague when we made this statement. We made this statement to relate to the case where λ_k are non-zero and decay at a certain rate. In such a case, this rate of decay controls the smoothness of the resulting GP. This is a nice result from spline theory.

To address your point, we have made this modification.
"We call this a \emph{bandlimited} spectrum with passband $[f_\mathrm{lo}, f_\mathrm{hi}]$ and bandwidth $f_\mathrm{hi} - f_\mathrm{lo}$. As the bandwidth increases, the receptive fields become less smooth since they are made up of a wider range of frequencies. ~~The smoothness is also controlled by the overall magnitude of the nonzero eigenvalues.~~ If the λ_k are all nonzero but decay at a certain rate, this rate controls the smoothness of the resulting GP \cite{wahba1990}."

10. In describing the computation of C_data in Section 2.2.2, an explanation of what is meant by 'centered receptive fields' could be helpful.
Thank you for catching that. The "centered receptive fields" doesn't make sense in the context of mechanosensors. It only makes sense for V1 type receptive fields.

1. **To address your point, we removed it from Section 2.2.2.**
   "To characterize the receptive fields of this population of CS neurons, we compute the data covariance matrix $\mat C_{\rm{data}}$ by taking the inner product between the ~~centered~~ receptive fields. "

11. In Section 2.2.3, the unit pixels^2 seems incorrect, since a squared pixel is what is generally meant by a pixel.
Thank you! We fixed that in the text everywhere we mention "pixel^2" to "pixel."

12. More explanation of what mechanism leads to the prominent dark patches in the covariance matrices shown in Figure 5 would be appreciated.

From equation (8), the covariance function has the following form:

$$C(t,\ t') \ = \ exp(\frac{-|t-t'|^2}{2\,f^2}) \cdot exp(\frac{-|t-c|^2 \ -|t'-c|^2}{2\,s^2})$$

The formation of dark patches is primarily caused by the localization term $exp(\frac{-|t-c|^2 \ -|t'-c|^2}{2\,s^2})$. The covariance between any two pixels t and t' decays as a function of their distance from the center, c, of the receptive fields. So, pixels closer to c will have larger covariance with each other. When we flatten the image as a vector to calculate the covariance between pixels, this will show up as regular dark patches. The magnitude of s controls the number of the dark patches. On the other hand, the diagonal structured comes from the spatial frequency term, $exp(\frac{-|t-t'|^2}{2\,f^2})$. The covariance also decays as a function of the distance between pixels. If we have a larger f, the correlation between farther pixels is larger. This shows up as a larger number of off diagonals.

To address your point, we made this change in Section 2.2.3 Para 10.
"In the covariance model, the number of off-diagonals, the center, the rate of their decay away from the center are determined by the parameters \$f\$, \$s\$ and \$\vec{c}\$ respectively. The covariance between pixels decays as a function of their distance from \$\vec{c}\$. This leads to the the equally-spaced non-zero segments. On the other hand, the covariance also decays as a function of the distance between pixels. This brings the the diagonal structure to the model. When the frequency parameter \$f\$ increases, the number of off-diagonals increases. Pixels in the generated weights become more correlated and the weights become spatially smoother. When the size parameter \$s\$ increases, the diagonals decay slower from the center \$\vec{c}\$, increasing correlations with the center pixel and leading the significant weights to occupy more of the visual field."

13. For the result in Figures 5E (and, to an extent, in Figure 3A), what do the authors mean by 'remarkably well'? This might be addressed by the response to major point 1. For Figure 5E, can the authors comment on the faster decay in the spectrum of the model? Could this be due to noise or some effect related to the calcium imaging?

Thank you for the question. We hope that our response to the major point 1 suffices for quantifying our claim of fitting the data "remarkably well".

As to the second question, the spectrum of the model decays faster than the data because of discretization. The model eigenvalues were calculated numerically from the covariance matrix on a grid that matched the receptive field pixel dimensions. When we derive the explicit form of the eigenvalues in continuous space, we see a good match between the data and the analytical

eigenvalues (shown in Figure 5E as “analytical”) up to 80 eigenvalues, which explain 86% of the variance in the data. However, even the analytical eigenvalues decay faster than the data after around 80 eigenvalues. We believe that this is due to noise because the corresponding data eigenfunctions do not exhibit much structure and look noisy.

To address your point, we added a short snippet to clarify this in the last paragraph of Section 2.2.3.
“Similar spectral properties are evident in the eigenvectors and eigenvalues of $\mat C_{\mathrm{model}}$, $\mat C_{\mathrm{finite}}$, $\mat C_{\mathrm{data}}$, and the analytical forms derived in \eqref{eq:V1_eigen} (Fig.~\ref{fig:cov_V1}D,E). As in Section~\ref{sec:mechanosensors}, the covariances are normalized to have unit trace.
Note that the analytical eigenfunctions are shown on a finer grid than the model and data because the analysis was performed in continuous space. The differences between the eigenfunctions and eigenvalues of the analytical and model results are due to discretization.
Examining the eigenvectors (Fig.~\ref{fig:cov_V1}D), we also see a good match, although there are some rotations and differences in ordering. These 10 eigenvectors explain 68$\%$ of the variance in the receptive field data. For reference, the top 80 eigenvectors explain 86$\%$ of the variance in the data and all of the variance in the model. The eigenvalue curves of both the models and the analytical forms match that of the data (Fig.~\ref{fig:cov_V1}E) reasonably well, although not as well as for the mechanosensors. In Appendix~\ref{app:cov_v1}, we repeat this analysis for receptive fields measured with different stimulus sets in the mouse and different experimental dataset from non-human primate V1. Our findings are consistent with the results shown above.”

14. In the text describing Figure 5A, it is stated that C_data is shown zoomed in, but nothing about this is mentioned in the caption, and no mention of the scale is given, so that one has to look at the supplemental figure to get some (still imprecise) idea of what's going on. Given that the dark patches are still clearly visible in the full covariance plotted in the supplemental figure, I would recommend either showing the full version instead in Figure 5 or describing the zoomed-in version more clearly.

Thank you for this point. The full covariance matrix is 504 x 504 pixels Its non-zero portion is the 180 x 180 pixel region around the center. This is what we mean by the “zoomed in” version.

To address your point, we add this clarification in the Section 2.2.3, Paragraph 6.
“We compute the data covariance matrix $\mat C_{\rm{data}}$ by taking the inner product between the receptive fields. We normalize the trace to be the dimension of each receptive field, which in this case is (14 $\times$ 36) pixels = 504 pixels. The data covariance matrix resembles a tridiagonal matrix. However, the diagonals are non-zero only at equally spaced segments. Additionally, their values decay away from the center of the matrix.
We show $\mat C_{\rm{data}}$ zoomed in at the non-zero region around the center of the matrix (Fig.~\ref{fig:cov_V1}A); this corresponds to the 180×180 pixel region around the center

of the full 504×504 pixel matrix. The full covariance matrix is shown in the Appendix \ref{app:cov_v1} (Fig.~\ref{fig:cov_V1_whitenoise_full}).”

We also add this clarification in the caption of the supplemental figure, Figure 19.
“We show the full structure of the covariance matrices shown in Fig.~\ref{fig:cov_V1}, which are the 180×180 pixel region around the centers of these 504×504 pixel matrices. These matrices are generated from the (A) receptive fields of 8,358 mouse V1 neurons, (B) the GP model \eqref{eq:cov_V1}, and (C) 8,358 random samples from the model.}”

15. In the plots for the machine learning applications, why did the authors add the green curves? The fact that they show higher test errors than the blue ones is tautological, since the parameters for the latter were optimized precisely to minimize this test error. There may be some value in these curves if the point is illustrate that a poorly chosen inductive bias is worse than no inductive bias at all, but this doesn't seem to be borne out by all pairs of green vs. orange curves in the figures, and this doesn't seem to be the point that is made in the text.

Thank you for the question. The reason we add the green curves is exactly your reasoning. We want to show that a poorly chosen inductive bias is worse than no inductive bias at all. In the Figures 6 and 7, where we use random features networks i.e. hidden layer is not optimized but only the output layer is, we do see that the poorly chosen inductive bias is worse than no inductive bias at all. The training error is greater for “incompatible” networks than the unstructured networks.

However in Figure 7, where we jointly optimize the hidden and output layers, the training and testing error for unstructured and incompatible networks look the same at the end of training. This is because the optimization procedure eventually overcomes the poor inductive bias. But notice around the beginning of training, the network with poor inductive bias starts off with a larger loss compared to the unstructured network. There is still an effect of poor inductive bias for these networks.

We added the following sentence to the end of section 2.3.5:
“Also, in many of these scenarios the incompatible structured weights get to performance on par with the compatible ones by the end of training, when the poor inductive bias is overcome.”

16. In the last paragraph of appendix A.1, the authors claim that 'functions with small H-norm are easier to learn than those with larger norm'. Can this statement be made more precise, perhaps for a specific regression algorithm?

Great point! Yes this statement can be made more precise. Firstly, when we say that “functions with small H-norm are easier to learn” we mean in the precise sense that it takes a smaller training set size to achieve a given generalization gap i.e. the difference between the training and testing error. This gap is proportional to the H-norm of the function. Under certain assumptions, this gap for regression using mean squared loss is directly proportional to the square of norm of the function, $|| f||_H^2$, and inversely proportional to the number of training data. We refer to Theorem 3.9 in Shawe-Taylor and Cristianni for the full theorem.

To address your point, we have added a new section in the appendix where we go over this theory of norm comparison in detail. In Section A.2, we analyze the target function norm for both structured and unstructured networks for a simplified version of the frequency detection example. We show that indeed the target function’s norm is smaller in the RKHS of the structured network. We expand on the relationship between the generalization gap and the function norm in the first paragraph of Section A.2.5.

“**Section A.2.5: Comparison of the learning performance in structured and unstructured RKHS**

A very standard but rough bound on the generalization performance of kernel ridge regression or classification can be found by analyzing the Rademacher complexity of the class of linear functions in the RKHS \citeapp{Shalev-shwartz_ben-david_2014, shawe-taylor_cristianini_2004}. These upper bound the expected loss over new data in terms of the training loss plus an error term, c.f.\ Theorem 7.39 in \citeapp{shawe-taylor_cristianini_2004} for kernel ridge regression. The error term controls the generalization gap between test and training losses and is typically proportional to the $\| f^* \|_\mathcal{F}$ (for 0/1 loss) or $\| f^* \|_\mathcal{F}^2$ (for square loss).\footnote{The trace of the kernel matrix also appears in these bounds, but this is unity due to normalization \eqref{eq:kernel_trace_normalize}.} Thus, a function with small $\mathcal{F}$-norm is {\em easier to learn} in the {\em precise sense} that it takes a smaller training set size to achieve a given generalization gap when the norm is smaller. “

Reviewer 3:

1. The theoretical analysis on the equivalence between structured weight vector transformation and filtering into Gaussian Processing eigenbasis and doing a random projection onto a spherical random gaussian is interesting. Aside from its connection to the kernel theory of learning, what are the broader implications of this finding, either in sensory neuroscience or deep learning fields? It would be helpful to get more discussion around this topic in a revised version.

Thank you for a good question. The theoretical analysis does have broader implications for both sensory neuroscience and deep learning, as you suggest.

For sensory neuroscience, this GP approach can be easily applied to model auditory, somatosensory, and tactile regions and understand the basis change done by these systems. **We discuss these ideas in Discussion Section 3.1.**

There are also exciting new directions in terms of connecting this GP approach to development. In particular, it would be interesting to understand what kind of developmental mechanisms might give rise to structured but random receptive fields. **We comment on this connection to development in Discussion Section 3.2. We highlight possible future directions in Section 3.5, Paragraph 3.**

The connection to kernel learning relates our work to deep learning. We can also study how task driven learning that changes all weights (backpropagation) affects this basis change idea by studying the limiting neural tangent kernel (NTK). The NTK is slightly different from the kernels that we considered. As the neural networks increase in width, they converge to a deterministic kernel machine. This is a major technique used to study the learning properties of neural networks. Recent work by Dapello et. al. showed that CNNs with V1-like convolutional layers at the front are robust to adversarial input perturbations. Our theory may help explain this robustness as filtering out high frequency corruptions. We also establish a connection between our work and wavelet scattering transform, which is a multi-layer network where wavelet coefficients are passed through nonlinearities similar to deep CNNs. Our framework is a randomized model and yields wavelets of single scale. **We discuss these connections to deep learning in Section 3.4, Paragraph 3 and Section 3.5, Paragraph 3**. We also added the following sentence (highlighted in green) to Section 3.5, Paragraph 3 to discuss the connection of our work to wavelet scattering networks:

“The random V1-inspired receptive fields that we model can be seen as similar to what happens in a convolutional neural network (CNN) \cite{Olah_2017}, which have similarities and differences compared to brains \cite{lindsay_convolutional_2020}. A recent study showed that CNNs with a fixed V1-like convolutional layer are more robust to adversarial perturbations to their inputs \cite{Dapello_2020}. In a similar vein to our work, using randomly sampled Gabor receptive fields in the first layer of a deep network was also shown to improve its performance

\cite{Illing_2019}. The wavelet scattering transform is a multi-layer network where wavelet coefficients are passed through nonlinearities, a model which is similar to deep CNNs \cite{mallat_group_2012, bruna_invariant_2013, anden_deep_2014}. Our framework differs as a randomized model and yields wavelets of a single scale, and similar studies of robustness and learning in deep networks with our weights are possible. Adding layers to our model or sampling weights with a variety of spatial frequencies and field sizes
would yield random networks that behave similar to the scattering transform, offering another connection between the brain and CNNs. Directly learning filters in a Hermite wavelet basis led to good performance in ANNs with little data \cite{Jacobsen_2016}, and this idea was extended to multiple scales by \cite{Pintea_2021}. Our structured random features can be seen as an RFN version of those ideas with supporting evidence that these principles are used in biology.”

2. What is the motivation for using "Gaussian Process" type generative model here, from mathematical and/or neuro- perspectives? Is it possible that sampling random filters from Gaussian Process is not only mathematically tractable, but also more biologically plausible (compared to, say, learning filters from the task-driven optimization)? It would be helpful to get more discussion around this in a revised version.

The mathematical motivation for the GP generative model is the clear connection to kernel learning theory. This connection opens the door to using techniques which are a mainstay in machine learning theory literature, like estimating generalization error and sample complexity, in the context of learning in biologically realistic networks. We expanded the discussion of this and gave more detailed theory in Appendix A.2 of the revision.

In terms of neuroscience motivation, we do look towards development and biological plausibility. We already know that unsupervised learning rules with local inhibition allows neurons to learn the principal components of their inputs. We conjecture that a modification of such learning rules could allow neurons to learn overcomplete, non-orthogonal projections of their inputs and give rise to structured but random receptive fields. Such a mechanism would certainly be more biologically plausible compared to task driven optimization. This is discussed in the Appendix and offers a good direction for future studies.

To address your point, we have added the following:

1. **In the Discussion (Section 3.2: Receptive fields in development):**
   “Our generative model offers new directions to explore the biological basis and computational principles behind receptive fields. Development lays a basic architecture that is conserved from animal to animal \cite{swanson_brain_2003,strausfeld_arthropod_2012}, yet the details of every neural connection cannot be specified \cite{Zador_2019}, leading to some amount of inevitable randomness at least initially \cite{Caron_2013}. If receptive fields are random with constrained covariance, it is natural to ask how biology implements this. Unsupervised

Hebbian dynamics with local inhibition can allow networks to learn principal components of their input \cite{oja_principal_1992,pehlevan_why_2018}. An interesting future direction is how similar learning rules may give rise to overcomplete, nonorthogonal structure similar to what has been studied here. This may prove more biologically plausible than weights that result from task-driven optimization."

2. **In the Discussion (Section 3.5: Limitations and future directions).**
"The random feature receptive field model is a randomized extension of the LN neuron model. The LN model fits a parameterized function to each receptive field \cite{Chichilnisky_2001}. In contrast, the random feature framework fits a distribution to an entire population of receptive fields and generates realistic receptive fields from that distribution. A natural question is how they compare. If the goal is to capture individual differences between neuronal receptive fields, one should resort to an LN model where each neuron's receptive field is fit to data. The random feature model is not as flexible, but it provides a direct connection to random feature theory, and it is mathematically tractable and generative. This connection to kernel learning opens the door to using techniques which are a mainstay in machine learning theory literature, for instance to estimate generalization error and sample complexity \cite{shawe-taylor_cristianini_2004}, in the context of learning in more biologically realistic networks."

3. **In the Introduction:**
"Given the stimulus presented and neural response data, one can then estimate the receptive fields of a population of neurons. Simple visual receptive fields have classically been understood as similar to wavelets with particular spatial frequency and angular selectivity \cite{Jones_Palmer_1987}. In mechanosensory areas, receptive fields are selective to temporal frequency over a short time window \cite{Fox_Fairhall_Daniel_2010}. Commonly, parametric modeling (Gabor wavelets \cite{stringer2019high}) or smoothing (regularization, etc. \cite{park2011receptive}) are used to produce ``clean'' receptive fields. Yet, the data alone show noisy receptive fields that are perhaps best modeled using a random distribution \cite{Bonin_2011}. As we will show, modeling receptive fields as random samples produces realistic receptive fields that reflect both the structure and noisiness seen in experimental data. More importantly, this perspective creates significant theoretical connections between foundational ideas from neuroscience and artificial intelligence. This connection helps us understand why receptive fields have the structures that they do and how this structure relates to the kinds of stimuli that are relevant to the animal."

3. Is it surprising that filter samples from GPs with covariance from the real data match various properties of real neural populations? It might be helpful to have control models which fail to capture such biological fidelity (for example in Fig 3,4) in a revised version.

We would say that it is not surprising that the filter samples from GPs with covariance from the real data match the various properties of real neural populations. This is because we designed the GP covariance to capture the known properties of these receptive fields.

To address your point, we added some control models which fail to capture such biological fidelity for both mechanosensory sensilla and V1 neurons.

- **We added two null hypotheses for mechanosensory sensilla in Section A.4: the fourier model from Section 2.2.1 and the unstructured model. We show the covariance matrices and the filter samples below.**

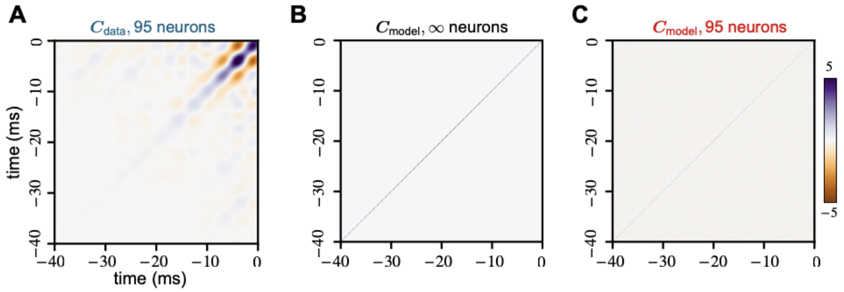

Figure 9: **Covariance matrix of mechanosensory receptive fields and unstructured model.** We compare the covariance matrices generated from the (A) receptive fields of 95 mechanosensory neurons, (B) unstructured GP model and (C) 95 random samples from the model.

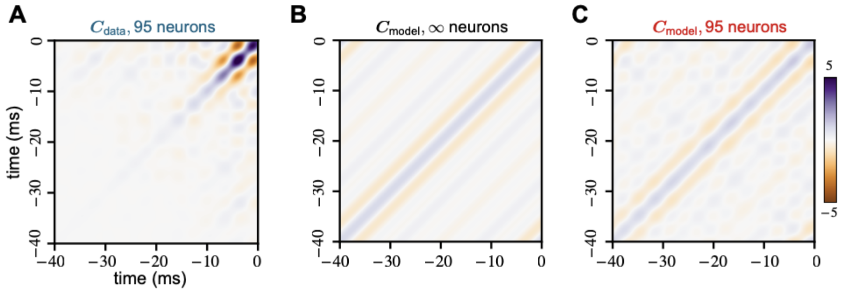

Figure 10: **Covariance matrix of mechanosensory receptive fields and the fourier model (5).** We compare the covariance matrices generated from the (A) receptive fields of 95 mechanosensory neurons, (B) fourier GP model and (C) 95 random samples from the model.

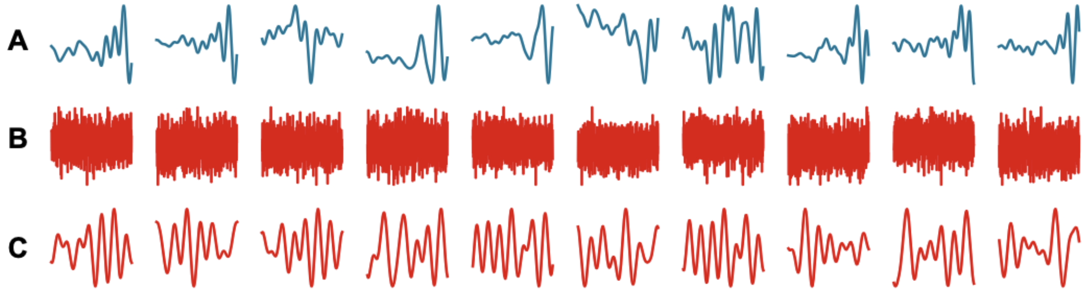

Figure 12: **Receptive fields from the unstructured model and the fourier model (5).** We show the receptive fields from the (A) mechanosensory neurons, (B) unstructured GP model and (C) the Fourier GP model.

- **We added two null hypotheses for V1 neurons. We show covariance matrices from two null V1 models in Appendix Section A.4: purely random receptive fields and translation invariant models of the existing RF model. In the translation invariant model, we remove the localization term from the covariance function in Equation 9. Now, the receptive field covers the entire visual field. We still fit the spatial frequency parameter. The equation for the covariance function is below.**

(Full model) $C(t,\ t') \ = \ exp(\frac{-|t-t'|^2}{2\,f^2}) \cdot exp(\frac{-|t-c|^2 + -|t'-c|^2}{2\,s^2})$

(Translation invariant model) $C(t,\ t') \ = \ exp(\frac{-|t-t'|^2}{2\,f^2})$

(Purely Random model) $C(t,\ t') \ = I$

The covariance matrices and sampled receptive fields for the null receptive field models of V1 are below.

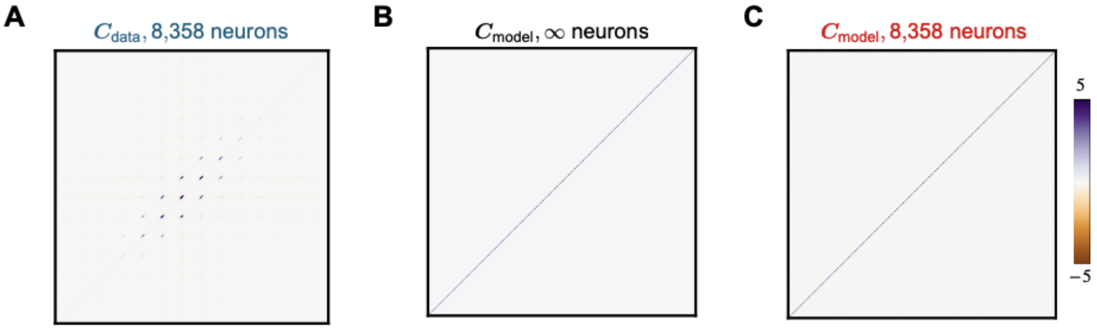

Figure 9: **Covariance matrix of V1 receptive fields and unstructured model for white noise stimuli.** We compare the covariance matrices generated from the (A) receptive fields of 8,358 mice V1 neurons, (B) unstructured GP model and (C) 8,358 random samples from the model.

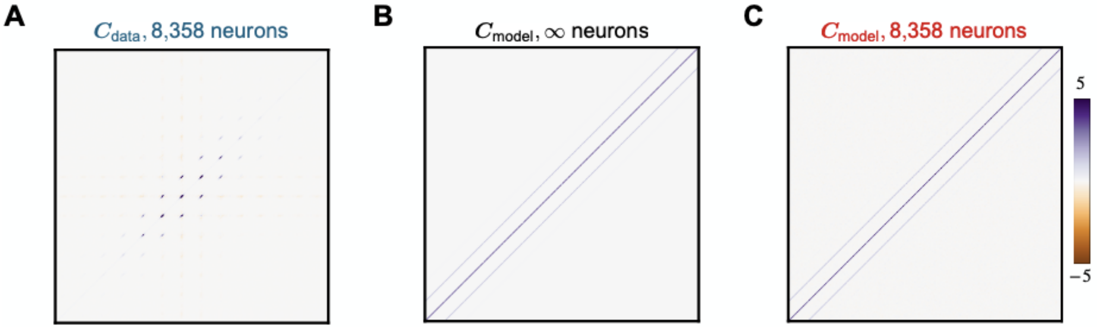

Figure 10: **Covariance matrix of V1 receptive fields and translation invariant V1 model (Eq. 15) for white noise stimuli.** We compare the covariance matrices generated from the (A) receptive fields of 8,358 mice V1 neurons, (B) translation invariant version of the GP model and (C) 8,358 random samples from the model.

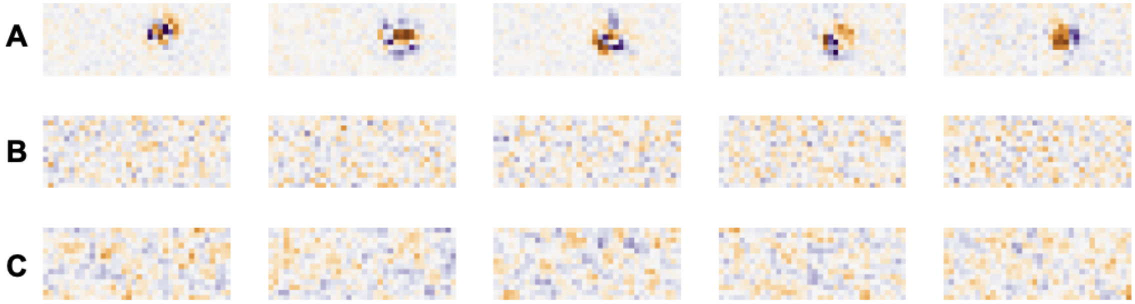

Figure 15: **Receptive fields from V1 neurons, the unstructured model and the translation invariant V1 model (28).** We show the receptive fields from the (A) V1 neurons, (B) unstructured GP model and (C) the translation invariant V1 GP model.

Minor comment: the name "structured random receptive field", while properly defined in the paper, is a bit vague — initially I thought that "random" here implies stochastic, as in Dapello et al.

To address your point, we added this sentence in the first paragraph of Section 2.1.1 Line 128 to clarify.

“We start by introducing the main learning algorithm and the neuronal model of our work, the RFN. Consider a two-layer, feedforward ANN. Traditionally, all the weights are initialized randomly and learned through backpropagation by minimizing some loss objective. In sharp contrast, RFNs have their hidden layer weights sampled randomly from some distribution and fixed. Each hidden unit computes a random feature of the input, and only the output layer weights are trained (Fig.~\ref{fig:network_architecture}). Note that the weights are randomly drawn but the neuron's response is a deterministic function of the input given the weights.”
